# Hairy root culture: a potent method for improved secondary metabolite production of Solanaceous plants

**DOI:** 10.3389/fpls.2023.1197555

**Published:** 2023-09-04

**Authors:** Diptesh Biswas, Avijit Chakraborty, Swapna Mukherjee, Biswajit Ghosh

**Affiliations:** ^1^ Plant Biotechnology Laboratory, Post Graduate Department of Botany, Ramakrishna Mission Vivekananda Centenary College, Kolkata, India; ^2^ Department of Microbiology, Dinabandhu Andrews College, Kolkata, India

**Keywords:** Agrobacterium rhizogenes, hairy root, Solanaceae, elicitation, metabolic engineering, bioreactor, solasodine

## Abstract

Secondary metabolites synthesized by the Solanaceous plants are of major therapeutic and pharmaceutical importance, many of which are commonly obtained from the roots of these plants. ‘Hairy roots’, mirroring the same phytochemical pattern of the corresponding root of the parent plant with higher growth rate and productivity, are therefore extensively studied as an effective alternative for the *in vitro* production of these metabolites. Hairy roots are the transformed roots, generated from the infection site of the wounded plants with *Agrobacterium rhizogenes.* With their fast growth, being free from pathogen and herbicide contamination, genetic stability, and autotrophic nature for plant hormones, hairy roots are considered as useful bioproduction systems for specialized metabolites. Lately, several elicitation methods have been employed to enhance the accumulation of these compounds in the hairy root cultures for both small and large-scale production. Nevertheless, in the latter case, the cultivation of hairy roots in bioreactors should still be optimized. Hairy roots can also be utilized for metabolic engineering of the regulatory genes in the metabolic pathways leading to enhanced production of metabolites. The present study summarizes the updated and modern biotechnological aspects for enhanced production of secondary metabolites in the hairy root cultures of the plants of Solanaceae and their respective importance.

## 1 Introduction

Metabolites are the small molecules which are intermediates and end products of physiological metabolism. While primary metabolites are essential for survival, secondary metabolites are not. The absence of secondary metabolites does not immediately restrain the life of an organism, but impairs it to a large extent. They confer adaptive roles, by functioning as defensive compounds or signalling molecules in ecological interactions, symbiosis, metal transport, competition, etc. Secondary metabolite production is influenced by growth, tissue differentiation and cell or body development, and external pressures.

Plants possess almost a limitless ability to synthesize a wide variety of secondary metabolic compounds in different organs like leaves, shoots, roots, and flowers via different biochemical pathways, which protect themselves against biotic and abiotic stress (Wang et al., 2022; Sharma et al., 2023). These aromatic compounds, like tannins, terpenoids, alkaloids, flavonoids, phenols or their oxygen-substituted derivatives, are extensively being used as agrochemicals, dyes, insecticides, food additives and pharmaceuticals (Gantait and Mukherjee, 2021; Sharma et al., 2022). Their utilization for the production of pharmacologically important compounds has gained tremendous attention over past decades as many of these compounds like dopamine, artemisinin, paclitaxel, atropine and morphine are now established as a commercial drug and being synthesized directly or indirectly from the plants (Katyal, 2022).

Among all the secondary metabolites, terpenes are the most diverse type of compounds accumulated by plants through five-carbon intermediates of isopentenyl diphosphate (IPP) and dimethyl allyl diphosphate (DMAPP) (Ruzicka, 1953). Another important group is alkaloid, composed of the nitrogen-containing heterocyclic compound. These nitrogen-containing compounds are mainly biosynthesized from amino acids like tryptophan, tyrosine, phenylalanine, lysine, and ornithine. There are diverse alkaloids found in nature, such as steroidal, pyrrole, pyrrolidine, tropane and indole alkaloids. Indole alkaloids are the largest group of alkaloids found in plants with indole skeletons (Hesse, 2002; Mehrotra et al., 2018).

As medicinal plants are the exclusive sources of these beneficial bioactive compounds, they are disappearing at a high speed. It has been found that, more than 95% of the medicinal plants are still being collected from the wild to meet the requirement of secondary metabolites. Many parts of the plants like leaves, bark, roots, fruits, seeds or even the whole plant are indiscriminately being collected from the wild habitats, without taking care of the mother plants. Due to this over-exploitation and habitat destruction, many of these important useful species are on the verge of extinction. So, there is a clear need to encourage multiplication of these plants in an alternative way. It must also be noted that the content of secondary metabolites in wild habitats of the plants is highly dependent on multiple seasonal and environmental factors and not sufficient to meet the industrial demand. For these reasons, different *in vitro* systems like plant cell and organ cultures, immobilized plant cultures, transformed cultures, bioreactor cultures are now being utilized for enhanced synthesis of these bioactive compounds with elicitation, precursor feeding and biotransformation to facilitate an optimized production of tailor-made molecules (Espinosa-Leal et al., 2018; Habibi et al., 2018).

Plant cell and organ cultures can be established in the laboratory using explants like plant leaves, stems, roots, meristems, etc., for mass production of the whole plant as well as the required tissue. For extraction of secondary metabolites, organ culture or unorganized (callus or suspension) cultures are established depending upon the site of synthesis of the compound (Isah et al., 2018). For example, withanolide, is found in the roots of different Solanaceous plants. So, root cultures of these plants have been established to get a steady supply of the compound (Sivanandhan et al., 2021). Plant tissue culture methods provide a large number of regenerated plants (or tissue) in a short period of time, in a small area with high level of clonal stability (Chakraborty et al., 2022) and thus, a very useful biotechnological tool to produce secondary metabolites.

Secondary metabolite production through hairy root culture, obtained via *Agrobacterium rhizogenes-*mediated gene transfer techniques, has drawn much attention in last few decades. Hairy root system is a convenient and viable approach to get the target metabolites. They possess some extraordinary qualities that make them extremely advantageous for production of the secondary metabolites for example, huge biomass production in a short period of time and autotrophy in plant growth hormone (Yousefian et al., 2018). The ‘hairy root’ is nothing but a disease syndrome which results due to the invasion of T-DNA of the bacteria *Agrobacterium rhizogenes* into the plant genome, during infection. Due to their distinctive properties for enhanced secondary metabolite production, higher growth rate, genetic, biochemical and physiological stability, the hairy roots are chosen as a model system for the production of bioactive compounds over naturally grown plants (Yadav et al., 2020). Different secondary metabolites like alkaloids, flavonoids, and terpenes, are found in higher quantities in the hairy roots of genetically transformed plants of Solanaceae. Enhanced production of tropane group of alkaloids (the most abundant alkaloid found in these plants) have been reported previously in the hairy roots of many species of this family like in *Anisodus acutangulus, Anisodus tanguticus, Atropa baetica*, *Duboisia myoporoides*, *Hyoscyamus senecionis, Scopolia japonica* (Mano et al., 1986; Deno et al., 1987; El Jaber-Vazdekis et al., 2008; Kai et al., 2011; Dehghan et al., 2017) (**Supplementary Table 1**). These alkaloids include hyoscyamine, scopolamine, anisodine, atropine, anisodamine and cuscohygrin are well known for their extensive bioactivities. Some polyphenols with anti-microbial, anti-oxidant, anti-diabetic, anticancer and anti-inflammatory activities, are also reported to be found in the hairy roots of Solanaceae species. Polyphenols like cyanidin, peonidin derivatives, kaempferol derivatives and quercetin derivatives are found in the hairy roots of *Petunia hybrida, Nicotiana tabacum, Solanum lycopersicum, Solanum melongena* (Chen et al., 2012; Mishra et al., 2012; Tusevski et al., 2013; Anton et al., 2017). Bioactive terpenes and steroids are also found in the hairy roots of a few species of Solanaceae, including *Nicotiana tabacum*, and *Withania somnifera* respectively (Ritala et al., 2014; Rogowska and Szakiel, 2021). Presence of these bioactive compounds in the hairy roots of solanaceous plants make them commercially valuable and beneficial for mankind (Roy, 2021).

Hairy root cultures are efficient and advantageous as bioproduction systems for biosynthesis of any plant derived molecules or recombinant protein, and is also important for understanding the process of phytoremediation (Gutierrez-Valdes et al., 2020). In the last two decades, the ‘hairy root’ cultures have been investigated through different approaches, like genetic engineering of secondary metabolism pathways, increasing accumulation and excretion of secondary metabolites using elicitation and precursor feeding, production of pharmaceutically important recombinant proteins, and scaling-up of the culture process using bioreactors etc. (Rawat et al., 2019).

Following recent advancement in molecular biology, such as the CRISPR/Cas9 technology, high yielding lines have been developed for improved production of functional and efficient molecules, promising to food, cosmetic, and pharmaceutical industries (Häkkinen and Oksman-Caldentey, 2018; Gutierrez-Valdes et al., 2020). CRISPR/Cas9 strategy has been first utilized for production of eGFP in tomato hairy roots (Ron et al., 2014). α-solanine-free hairy roots of potato were successfully generated by genome editing of the *St16DOX* gene using CRISPR/Cas9 technology (Nakayasu et al., 2018). Gene editing and mutagenesis studies has also been carried out using CRISPR/Cas9, in *Brassica carinata*, potato, and soybean hairy roots respectively (Gutierrez-Valdes et al., 2020; Alok et al., 2023). Novel computational tools including modelling, neural networks, and artificial intelligence, can also result in improved yields (Häkkinen and Oksman-Caldentey, 2018).

The present study aims to describe the optimized production of different secondary metabolites through hairy root cultures, with reference to the plants of the family Solanaceae and to highlight different production improvement strategies to readily grasp and glance through the importance of hairy roots.

## 2 The family Solanaceae

Solanaceae is one of the largest plant family comprising over 100 genera and more than 3000 species worldwide (Morris and Taylor, 2017). The family of Solanaceae is known as nightshade and is found widely in India, Africa, and America. The family is enriched with many well-known genera, including *Solanum*, *Withania*, *Capsicum*, and *Physalis* which are good sources of food and food additives (Afroz et al., 2020). Many species of this family are consumed as the primary food and many also demonstrate medicinal properties. In ancient ethnopharmacology, many drugs were found to be derived from this plant family to treat different ailments. Various therapeutic compounds have been reported by different researchers which are used in modern medicine to cure deadly diseases. Many species show a wide range of antimicrobial, anti-inflammatory, anti-rheumatic, anti-septic, anti-hemorrhoidal activities. Many members are also used to treat dermatitis, orchitis, arthritis, headaches, infections, sedatives, chronic fatigue, weakness, premature ageing, emaciation, debility and muscle tension (Afroz et al., 2020). To date, extensive studies have been done on the secondary metabolites fingerprinting of plant species under Solanaceae. The pharmacological effects of the plants are greatly attributed to the secondary metabolites including terpenes, cardiac glycosides, carotenoids, sterols, phenolics, phenolic acids, coumarins, flavonoids, tannins, lignin, and nitrogenous compounds including alkaloids and glucosinolates (Chowański et al., 2016). Alkaloids are the most common secondary metabolites found in the plants of Solanaceae species. They are used as medicine by various tribes of people, and in traditional medicine systems, including Ayurveda, Traditional Chinese Medicine (TCM), Siddha, Unani, and homoeopathy. The alkaloids found are also proven for their application as antimicrobial, insecticidal, anti-infectious agents, and poisonous activity (Shah et al., 2013; Chowański et al., 2016). The compounds like α-tomatine, α-chaconine, and α-solanine, found in some members of the plants of Solanaceae, are proven for their anti-insecticidal activity (Chowański et al., 2016) and can be used as bio-insecticide to protect other plants prone to insecticidal attack. Among different alkaloids, tropane alkaloids are the signature alkaloids found in this family. These andglycoalkaloids, pyrrolizidine, and indole alkaloids, protect the plant itself against insects, herbivores and predators’ attack.

Some major secondary metabolites that are found in solanaceous plants include, tropane alkaloids, atropine predominantly found in *Atropa belladonna*, scopolamine in *Datura* spp., *Hyoscyamus niger*, and *Brugmansia* spp., hyoscyamine in *Hyoscyamus niger* and *Datura* spp. There are also some other alkaloids found in different plant species of Solanaceae like nicotine is often found in *Nicotiana tabacum*, and *Datura* alkaloids in *Datura* spp. and *Brugmansia* spp. The glycoalkaloids α-solanine and α-chaconine are reported in *Solanum tuberosum*, whereas solasonine and solamargine in various *Solanum* spp. Some important steroidal glycoalkaloids like tomatine and solasodine are reported to be found in *Solanum lycopersicum* and other *Solanum* spp. respectively. Withaferin A, Withanolide A, B, and D are the predominant steroidal lactones that are found in *Withania somnifera* and other related solanaceous species. Few phenolics compounds are also found in solanaceous species including capsaicin (*Capsicum* spp.), chlorogenic acid, rutin, caffeic acid (*Solanum lycopersicum* and other *Solanum* spp). *Solanum virginianum* is well known for the containment of triterpenoids and steroids like β-sitosterol. Another important group of compound coumarins, is also found in different *Solanum* species. Scopoletin is found in *Scopolia carniolica* and *Scopolia japonica* whereas, flavonoids like quercetin is found in *Solanum lycopersicum*.

## 3 *Agrobacterium rhizogenes* mediated transformation and hairy root biology

*Agrobacterium rhizogenes*, or currently *Rhizobium rhizogenes*, is a Gram-negative soil bacterium, that causes hairy root disease in higher plants under natural conditions (Rawat et al., 2019). Hairy roots are characterized by their high growth rate with lateral branching, profuse root hairs, and plagiotropic growth (Tepfer, 1984; Sevón and Oksman-Caldentey, 2002; Häkkinen and Oksman-Caldentey, 2018). However, the most interesting feature of hairy roots is their ability to accumulate secondary metabolites, following the same profile as the source plants, at much higher quantities compared to that of the parent plant or undifferentiated cell cultures (Häkkinen and Oksman-Caldentey, 2018). Stable production of metabolites for 5 to 16 years, in the same hairy root lines, have been reported in the long-term studies conducted in many plants like *Catharanthus roseus*, *Datura stramonium*, and *Hyoscyamus muticus*, (Maldonado-Mendoza et al., 1993; Peebles et al., 2009; Häkkinen et al., 2016).

**Figure 1:**
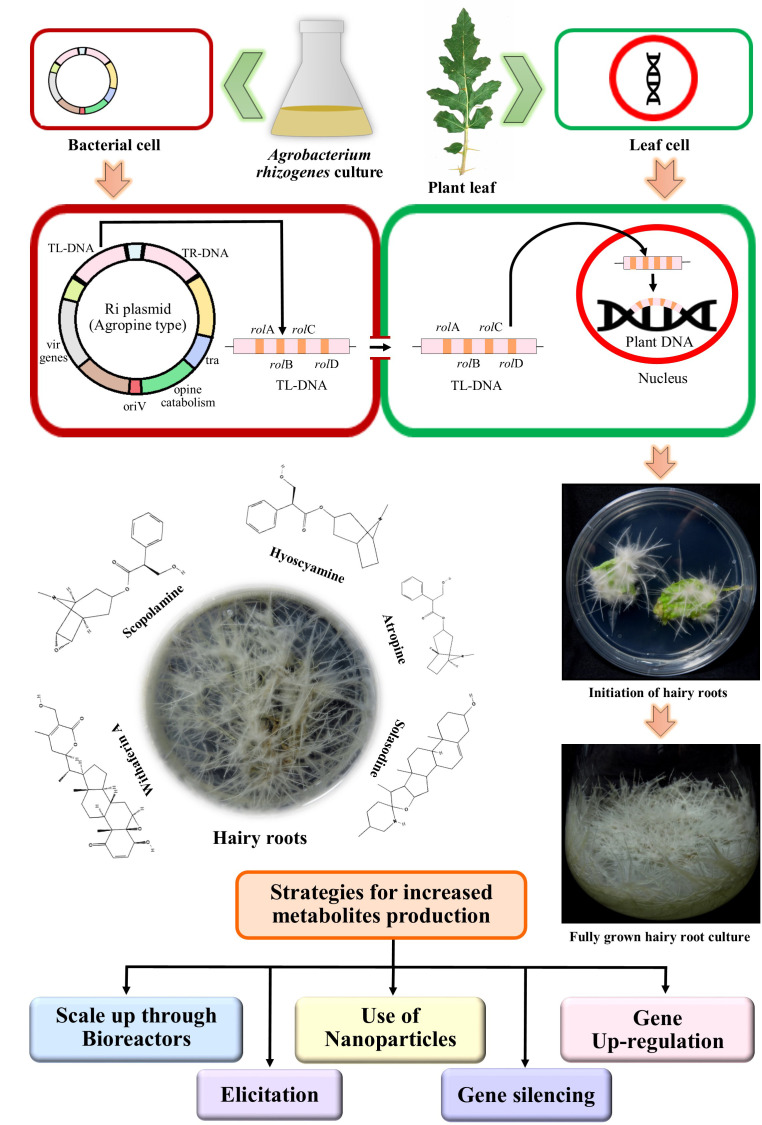
Outlined diagram of *Agrobacterium rhizogenes* mediated infection to the plant cell, induction and fully grown hairy root culture, producing bioactive compounds, and their enhancement strategies.

Infection process starts with the chemotaxis of the bacterium *A. rhizogenes*, to the wounded site of the plants, caused by insect attack or mechanical injury, producing simple phenolic compounds like acetosyringone (Giri and Narasu, 2000; Georgiev et al., 2007). The bacterium then invades the host plant tissue and transfers T-DNA (transfer DNA), segment of its root inducing plasmid (Ri-plasmid), with the help of the virulence (*vir*) genes of the Ri plasmid and the *chv* genes of the bacterial chromosomal DNA (**Figure 1**). T-DNA then gets integrated into the host plant genome (Dhiman et al., 2018). The T-DNA portion contains the genes for phytohormones like auxin and cytokinin synthesis, which causes the characteristic hairy root development and genes that code for opine synthesis, which are modified amino acids required for the nutrition of the bacterium (Georgiev et al., 2007; Srivastava and Srivastava, 2007).

It has been found that four of the 18 ORFs of the T-DNA of *A. rhizogenes*, are essential for hairy root formation, and termed as “*rol*” genes (*rolA*, *rolB*, *rolC*, and *rolD*), after “rooting locus” genes; these oncogenes modulate plant cellular processes, like phytohormone homeostasis, auxin and cytokinin metabolism, and signalling (Paolis et al., 2019; Gutierrez-Valdes et al., 2020). The gene *rolB* is the chief regulator of secondary metabolism, as it activates specific transcription factors of most explicit metabolic pathways. It has been found that about a 100-fold increase in resveratrol production occurred in *Vitis amurensis* when transformed with only the *rolB* gene (Paolis et al., 2019; Gutierrez-Valdes et al., 2020). *rolB* also hydrolyses bound auxins resulting in higher level of intracellular indole-3-acetic acid, which induces hairy roots (Rawat et al., 2019). Similarly, production of tropane alkaloids, pyridine alkaloids, indole alkaloids, and ginsenosides, can be stimulated by the actions of *rolC* gene alone (Faraz et al., 2020). RolC protein leads to major alteration in the metabolism of cytokins and gibberellins. In a study, done by Bonhomme et al. (2000) the *rolC* gene alone was found as efficient as all the *rolABC* genes for hyoscyamine and scopolamine production in hairy roots of *Atropa belladonna*. *rolC* actually hydrolyses cytokinin-conjugates liberating cytokinin, and have a cumulative effect on hairy root growth when expressed with *rolA* and *rolB* (Schmülling et al., 1988; OoNo et al., 1990; Estruch et al., 1991; Schmülling et al., 1993; Bahramnejad et al., 2019). Likewise, nicotine production was also reported to be stimulated by the actions of *rolA* gene alone (Faraz et al., 2020). *rol*A has a role in long-term cultivation of hairy roots and increased production of anthraquinones (Veremeichik et al., 2019; Gutierrez-Valdes et al., 2020).

## 4 Strategies adopted for enhancement of secondary metabolite production

Hairy root cultures are attractive systems for stable and continuous production of secondary metabolites with higher yields (Halder et al., 2018). However, in order to meet the demands of the commercial market, escalated production is necessary and a large number of investigations have been carried out on the scale-up strategies using different approaches (**Figure 1**).

### 4.1 Culture systems used for scaling up of hairy root production

Hairy root morphology demands specific designs of bioreactors suitable for hairy root culture. Unlike the cell cultures, hairy roots are sensitive to shearing and forms callus clumps, causing problems in mass transfer (Häkkinen and Oksman-Caldentey, 2018). Keeping in mind the structural features and specific requirements of hairy root cultures, different types of bioreactors like conventional airlift, bubble column, stirred tank, airlift balloon, and nutrient mist bioreactors are developed. The bioreactors used for hairy root cultures are mainly based on gas or liquid phase or combined gas and liquid phases (Dhiman et al., 2018). In gas-phase bioreactors, like in the mist reactors, the hairy roots remain exposed to air for proper oxygen supply, and nutrients are provided as droplets (Georgiev et al., 2013; Srikantan and Srivastava, 2018). Whereas, in liquid-phase bioreactors, like in stirred tank, airlift and bubble column reactors, liquid medium is used to submerge the roots to provide the nutrients (Doran, 2013). Disposable bioreactors such as, wave-reactors, have emerged as promising tools for hairy root culture systems as these do not require cleaning and sterilization and thus are cost-effective (Eibl et al., 2011; Lehmann et al., 2014; Gutierrez-Valdes et al., 2020).

Different culture systems such as shake flasks, bioreactors, air-lift reactors, and turbine blade reactors have been utilized for secondary metabolite production in hairy root cultures in different solanaceous plants like *Atropa belladonna*, *Duboisia leichhardtii*, and *Physalis minima* (Muranaka et al., 1992; Muranaka et al., 1993a; Muranaka et al., 1993b; Gansau and Mahmood, 2013; Habibi et al., 2015) (**Supplementary Table 1**). The hairy roots of *Anisodus tanguiticus* accumulated greater biomass, when the culture volume of flask reactors was increased (Lei et al., 2020). Bioreactor grown roots have been reported to produce higher amount of atropine than shake-flask cultures in *A. belladonna* hairy root cultures (Sharp and Doran, 1990). Similar result was found by Singh et al. (2021) in *A. belladonna*, where bioreactor upscaling has improved the yield of curcumin up to 2.3-fold compared to that of shake flask cultures. In *Brugmansia candida* a modified 1.5 L stirred tank was used to produce scopolamine and anisodamine from the hairy root cultures, which was proved to be the superior process in biomass production and alkaloid yield, compared to culturing in Erlenmeyer flasks (Cardillo et al., 2010). Stirred tank reactors were also used to maximize biomass accumulation and tropane alkaloid production in the hairy root culture of *Datura stramonium* (Hilton and Rhodes, 1990; Marchev et al., 2012). The growth rate and exudate concentration of *Hyoscyamus niger* hairy roots were higher when cultured in bioreactor, compared to shake flasks (Kareem et al., 2019). Anisodamine, scopolamine, hyoscyamine and cuscohygrine were reported to be produced in higher concentrations, when bubble-column bioreactor and a hybrid bubble-column/spray bioreactor were used in an effort to produce tropane alkaloids in *H. niger* hairy root cultures (Jaremicz et al., 2014). In a study in *Solanum chrysotrichum* hairy roots by Caspeta et al. (2005), the production of antifungal saponins were increased from 0.04% dry weight in 250 mL flasks, to 0.7% dry weight in 2 L of modified draught-tube internal-loop airlift reactors, which is about six times higher than the plant leaves. This validates the use of bioreactors as an excellent and adequate tool for improved metabolite productions.

### 4.2 Optimization of media components for secondary metabolite production

Media components, that is, concentration of nitrogen, phosphorus, carbon source and pH are important factors for both growth and secondary metabolite production in hairy roots. Different ions, such as NO_3_^–^, NH_4_^+^, KHPO_4_^-^, Ca^2+^ and Mg^2+^ can be utilized for manipulating secondary metabolite synthesis. Tropane alkaloids are synthesized from polyamine precursors; thus, tropane alkaloid biosynthesis can be boosted by increasing the concentration of nitrogen sources. Using 90 mM nitrogen (NH_4_^+^/NO_3_^–^ = 4:1), the highest cell yield (4.5 g/L) and the maximum alkaloid production (9.9 mg/L) was achieved in *Anisodus acutangulus* hairy roots (Liu et al., 2013). Increasing the NO_3_^–^ concentration in the medium also increased the alkaloid concentration and hyoscyamine/scopolamine ratio (Chashmi et al., 2010). The inclusion of NO_3_^–^ to the B5 medium before culturing, is also suggested by Amdoun et al. (2009), for better metabolite productions. Sáenz-Carbonell and Loyola-Vargas (1997) have observed the stimulation in alkaloid excretion in *Datura stramonium* hairy roots, when NH_4_^+^ was used as a sole nitrogen source. Then again, Praveen and Murthy (2013) have observed better production of withanolide A in *Withania sominifera* hairy roots when, concentration of NO_3_^–^ was much higher than NH_4_^+^. Lower concentration of Ca^2+^ in the medium of *D. stramonium* hairy roots has also reduced the hyoscyamine synthesis (Piñol et al., 1999). Higher concentrations of Mg^2+^ has also supported the greater productions of hyoscyamine and scopolamine (Hank et al., 2003). Harfi et al. (2011) suggested the use of diluted media (½MS, ¾MS, ½B5 and ¾B5) for hyoscyamine production. The use of sucrose as carbon source increased the production of alkaloids and withanolide A in hairy root cultures of *A. acutangulus and W. sominifera* (Praveen and Murthy, 2012; Liu et al., 2013). Again, withanolide A production was higher in pH 6.0 while pH 4.5 is optimum for alkaloid production (Praveen and Murthy, 2012; Liu et al., 2013). Pavlov et al. (2009) suggested the use of modified MS medium with 1.10 g/L KNO_3_, 0.17 g/L KH_2_PO_4_, and 40 g/L sucrose for diploid, and 50 g/L sucrose, for tetraploid *Datura stramonium* hairy root cultures, for optimal hyoscyamine production. Thus, it can be said that, media optimization is a crucial step in secondary metabolite production from hairy root cultures.

### 4.3 Application of elicitors for enhanced production of secondary metabolites in hairy root culture

Plants produce secondary metabolites as a defense response to several biotic and abiotic stresses, such as pathogen infection and environmental fluctuations. The agents that trigger plant immune response by activating signal cascade are referred as elicitors; they result in increased synthesis and accumulation of secondary metabolites. In general, elicitors act as signal molecules, rather being physiological effectors (Kai et al., 2018). Elicitation is a well exploited method for production of secondary metabolites in hairy root cultures and cell suspension cultures (Zhao et al., 2005; Wang and Wu, 2013). The activation of secondary metabolism is a result of complex interactions between the plant tissue and the elicitor used, and the response depends on several factors like elicitor specificity and concentration, time period of elicitation, and the particular developmental stage of the plant tissue (Zhai et al., 2017). Elicitors bind to the specific high affinity receptors present on the plant cell membrane, and reduces the protein electrochemical gradient by hindering ATPase activity, thus secondary intercellular messengers are generated, which ultimately transduces the defense response signals (Kaur et al., 2021).

Elicitors can be categorized as exogenous elicitors and endogenous elicitors, based on their origin. Exogenous elicitors are compounds that are foreign to the host plant, for example, the microbial peptides, glycoproteins, polysaccharides, polyamines, fatty acid components etc.; whereas endogenous elicitors are intercellular secondary messengers or signaling molecules synthesized within the host plant cell like salicylic acid, acetyl salicylic acid, jasmonic acid, methyl jasmonate, systemin etc. (Halder et al., 2019). Elicitors can also be categorized as biotic or abiotic type. Biotic elicitors have plant or microbial origins and can be either crude extracts like fungal homogenate and yeast extracts, isolates of bacteria and viruses, or of defined chemical composition like pectin, chitin, chitosan, alginate, curdlan, xanthan, polysaccharides, and glycoproteins. Abiotic elicitors, on the other hand, are mainly physicochemical factors, including light and UV-radiation, temperature fluctuation, osmotic shock induced by sugar alcohols and salts like sorbitol, mannitol, sodium chloride, potassium chloride, and salts of heavy metals like silver nitrate, cadmium chloride, nickel sulphate, copper sulphate, vanadium sulphate etc. (Wang and Wu, 2013; Kai et al., 2018; Kaur and Pati, 2018; Halder et al., 2019).

Hairy root culture in itself is an extraordinary tool for the commercial production of varied group of secondary metabolites. Even so, the production levels can be further improved by the application of elicitors in the hairy root culture systems. Several studies have shown that enhanced production of different classes of metabolites can be achieved in hairy root cultures of solanaceous plants of *Anisodus acutangulus*, *Atropa acuminata*, *A. baetica*, *A. belladonna*, *Brugmansia candida*, *Datura metel*, *D. stramonium*, *Hyoscyamus albus*, *H. muticus*, *H. reticulatus*, *Solanum mammosum*, *S. myriacanthum*, *S. trilobatum*, and *Withania somnifera*, upon elicitation (Mehmetoğlu and Curtis, 1997; Kuroyanagi et al., 1998; Pitta-Alvarez and Giulietti, 1998; Jacob and Malpathak, 2005a; Jacob and Malpathak, 2005b; El Jaber-Vazdekis et al., 2008; Doma et al., 2012; Kai et al., 2012a; Shilpha et al., 2015; Ooi et al., 2016; Srivastava et al., 2016; Moharrami et al., 2017; Shakeran et al., 2017; Amdoun et al., 2018; Moradi et al., 2020; Fattahi et al., 2021) (**Supplementary Table 1**).

Solasodine production was studied in *S. trilobatum* by Shilpha et al. (2015), where the hairy root cultures have found to produce 3.3-fold increase in solasodine content compared to that of untransformed plant roots. The content was further increased by 1.9-fold by eliciting the cultures with 4µM methyl jasmonate. The combined effect of methyl jasmonate and β-cyclodextrin caused up to 12.46-fold increase in withaferin A content in hairy roots of *Withania somnifera* (Karami et al., 2023). Another intercellular signalling agent, salicylic acid, has been reported to enhance withanolide A (by 58-fold), withanone (by 46-fold), and withaferin A (by 42-fold) production in hairy roots of *W. somnifera* (Sivanandhan et al., 2013). Acetylsalicylic acid at a concentration of 0.1 mM have increased hyoscyamine and scopolamine content by 1.6 and 3.5-fold than that of the control, in hairy roots of *Hyoscyamus reticulatus* (Norozi et al., 2018). Jasmonic acid and aluminum chloride have amplified the release of hyoscyamine and scopolamine in hairy roots of *Brugmansia candida*, by about 1200% and 150% respectively (Spollansky et al., 2000). In *B. candida* hairy roots, elicitation with pectinase have also increased the release of scopolamine by 1500% and hyoscyamine by 1100% (Pitta Alvarez et al., 2003). Bacterial strains of *Pseudomonus* were used as biotic elicitors in *D. stramonium* hairy roots, which enhanced hyscyamine and scopolamine production maximally by 583% and 265% respectively (Moussous et al., 2018). Chitosan as a biotic elicitor has showed 2.5 to 3-fold enhancement in hyoscyamine production in hairy roots of *Hyoscyamus muticus* (Oksman-Caldentey et al., 1991). Improved production of tropane alkaloids by 1.51, 1.13 and 1.08-folds upon elicitation has also been reported in hairy root cultures of *A. acutangulus* when treated with ethanol, methyl jasmonate and Ag^+^ ion, respectively (Kai et al., 2012a) (**Supplementary Table 1**). Exogenous elicitors like CaCl_2_ (50 mM) and hemicellulose (0.5 U/mg) have also been reported to enhance the intracellular hyoscyamine and scopolamine accumulation (60–250%), release (60–200%) and production (45–200%) in hairy roots cultures of *B. candida* (Pitta-Alvarez et al., 2000). In another study, solasodine content was boosted up by 4-fold and 3.6-fold on the application of 100 mM and 200 mM NaCl (Srivastava et al., 2016). Elicitation with 1-2 g/L of NaCl has also produced three times more hyoscyamine in *Datura stramonium* hairy roots (Khelifi et al., 2011). Hence, elicitation can be a potent strategy for abundant supply of valuable secondary metabolites from hairy root cultures of solanaceous species, to meet broad spectrum demand of commercial production, without endangering the plants.

### 4.4 Use of nanoparticles for improved secondary metabolite production

Nanoparticles are particles with a size range of 1 to 100 nm and it has broad spectrum applications in the fields of health care, biomedical sciences, cosmetics, chemical industries, electronics, etc. (Chakraborty et al., 2022). Similar to biotic and abiotic elicitors, nanoparticles can trigger the production of secondary metabolites, although the underlying mechanisms are yet unknown. The use of iron oxide nanoparticles at 900 and 450mg/L have made five-fold increase in hyoscyamine and scopolamine content in hairy roots of *Hyoscyamus reticulatus* (Moharrami et al., 2017) whereas, 100mg/L of nano-zinc oxide caused upto 37% and 37.63% production of hyoscyamine and scopolamine in *Hyoscyamus reticulatus* hairy roots (Asl et al., 2019). Nanoparticle of silver has also been shown to be effective causing upto 2.42-fold increase of atropine production in hairy roots of *Datura metel* (Shakeran et al., 2015).

### 4.5 Metabolic engineering for improvement of secondary metabolite production in hairy root cultures

Besides elicitation, metabolic engineering is another promising approach to enhance secondary metabolite production in plant tissue cultures of medicinal plants. The complex metabolic pathways leading to the generation of a particular bioactive compound are generally composed of multiple steps, which are mostly enzyme regulated. Among these, some of them are rate limiting slow steps or steps that branch to another pathway. Thus, the enzymes involved in these steps act as key elements for the synthesis of that particular metabolite. Regulation of the expression of the genes for these enzymes, can lead to enhancement of product yields. In current times, metabolic engineering has become an attention seeking alternative for enhanced accumulation of secondary metabolites in medicinal plants, examples include *Salvia miltiorrhiza*, *Isatis indigotica* etc. (Kai et al., 2018). Metabolic engineering has been achieved in solanaceous hairy roots through:

#### 4.5.1 Overexpression of regulatory genes

Tropane alkaloids are a special class of secondary metabolites found in the solanaceous medicinal plants, of which hyoscyamine and scopolamine have particular pharmaceutical importance. Tropane alkaloid biosynthetic pathway starts with the *S*-adenosylmethionine (SAM) mediated *N*-methylation of putrescine to form *N*-methylputrescine. This step is catalyzed by putrescine *N*-methyltransferase (PMT, EC 2.1.1.53), and is the first committed step in this pathway. Tropinone reductase I (TRI, EC 1.1.1.206) regulates a critical metabolic branching point, specific to hyoscyamine, and thus is an important enzyme in the tropane alkaloid biosynthetic pathway. Lastly, hyoscyamine 6β-hydroxylase (H6H, EC 1.14.11.11) which convert hyoscyamine to 6β-hydroxyhyoscyamine through hydroxylation, and 6β-hydroxyhyoscyamine to scopolamine by epoxidation is the enzyme whose level of expression can affect the metabolite synthesis (Kai et al., 2012b; Cardillo et al., 2013). Several attempts have been made to overexpress *putrescine N-methyltransferase* (*PMT*), *hyoscyamine 6β-hydroxylase* (*H6H*) and *tropinone reductase I* (*TRI*) genes through metabolic engineering, for the improved production of the tropane alkaloids in hairy root cultures of different solanaceous species, including *Anisodus acutangulus*, *Atropa baetica*, *A. belladonna*, *Brugmansia candida*, *Datura metel*, *Hyoscyamus muticus*, *H. niger*, *H. reticulatus*, and *H. senecionis* (Jouhikainen et al., 1999; Moyano et al., 2003; Zhang et al., 2004; Zárate et al., 2006; Kai et al., 2009; Kai et al., 2011; Kai et al., 2012b; Liu et al., 2010; Wang et al., 2011; Yang et al., 2011; Cardillo et al., 2013; Dehghan et al., 2017; Shameh et al., 2021) (**Supplementary Table 1**).

The enzyme squalene synthase dimerizes two molecules of farnesyl diphosphate and produces squalene, a common precursor in steroid and triterpenoid biosynthetic pathways in plants. The *squalene synthase* gene of *Arabidopsis thaliana* was transferred in *Withania coagulans* using *Agrobacterium rhizogenes* A4 T-DNA, under the control of CaMV35S promoter. The engineered hairy roots showed increased capacity of phytosterol and withanolide production, compared to that of control transformed roots, devoid of *squalene synthase* gene (Mirjalili et al., 2011). The production of withanolide A, withanolide B, withaferin A and withanone have also been increased by the attempt of overexpressing *squalene synthase* gene in cultured hairy roots of *Withania somnifera* (Sivanandhan et al., 2020).

#### 4.5.2 RNAi mediated gene silencing

RNA interference or RNAi technology can be well utilized, for downregulation of enzymes, of competing pathways for the common precursors, thus in turn increasing the production levels of the metabolite of interest. In *Duboisia leichhardtii*, the *quinolinic acid phosphoribosyl transferase* (*qpt*) gene was downregulated by gene silencing through *QPT-RNAi* construct, to stop the resource partitioning to the competitive pyridine alkaloid pathway, and thus, the tropane alkaloid production was increased (Singh et al., 2018) (**Supplementary Table 1**). In another study by DeBoer et al. (2011) the enzyme ornithine decarboxylase of *Nicotiana tabacum*, responsible for nicotine production was downregulated through RNAi methodology. This resulted in reduced nicotine and increased anatabine production in cultured hairy roots of *N. tabacum*, despite of stimulating the wounds.

#### 4.5.3 Heterologous expression of *Vitreoscilla* hemoglobin

In plant systems, the expression of *Vitreoscilla* hemoglobin can increase the productivity of oxygen-requiring metabolic pathways, by increasing the oxygen delivery, under hypoxic conditions. The biosynthetic pathway of tropane alkaloid require oxygen in the final step. Targeted expression of *Vitreoscilla* hemoglobin in the plastids of hairy roots of *Hyoscyamus niger* promoted accumulation of hyoscyamine and scopolamine by 1.25 and 2.2 fold, respectively (Guo et al., 2018). Expression of *Vitreoscilla* hemoglobin has also been seen to promote hyoscyamine production in hairy roots of *Hyoscyamus muticus* (Wilhelmson et al., 2005).

## 5 Pharmacologically important secondary metabolites production in solanaceous hairy root culture

The plant family of Solanaceae produces many secondary metabolites which are of immense pharmacological importance (Hamill et al., 1986; Rhodes et al., 1986; Furze et al., 1987; Robins et al., 1987; Walton et al., 1988; Benson and Hamill, 1991; Jovanović et al., 1991; Fecker et al., 1992; Ikenaga et al., 1995; Zehra et al., 1998; Jualang Azlan et al., 2002; Okršlar et al., 2002; Komaraiah et al., 2003). Among these, alkaloids, and more specifically, tropane alkaloids are the ‘signature alkaloids’ and are found as major compounds in different genera of Solanaceae (Zhang et al., 2023a) Three main representatives of this group, hyoscyamine, atropine, and scopolamine whose production through hairy root culture have been investigated in detail. Hairy root cultures of two other important bioactive compounds of Solanaceae, namely, solasodine (a steroidal alkaloid) and withaferin A (one of the major withanolides and a steroidal lactone) have also been explored extensively for enhanced production of these compounds (**Figure 1**).

### 5.1 Atropine

Atropine is a tropane alkaloid, found in many solanaceous plants, including *Atropa belladonna*. Atropine is a compound with diverse bioactivity, as elaborated in many previous works of literature. Atropine can treat parasympathetic nervous system disease. The prevalence of the disease myopia has been high in recent days. About 30% of the global population is suffering from this disorder, and it is predicted that about 50% of the total population will suffer from this disease in 2050. Myopia is a health issue regarding eye sight-threatening complications and further can cause total blindness in humans. High myopia can cause the death of a human being. So, controlling the disease in the adult age is mandatory to save the life of infected people. A high dose of atropine (0.5%–1%) is reported, for recovery from the disease, but leads to some side effects like blurred vision, photophobia, tachycardia, flushing, and hot skin. Furthermore, a low dose-dependent treatment (0.01%) trial on humans with atropine is more effective in controlling this myopia with greater efficacy without any side effects. However, respiratory failure and death can also be caused by excessive atropine treatment in humans. Summarizing from the basics in pharmacology, atropine works by inhibiting postganglionic acetylcholine receptors and direct vagolytic action (McLendon and Preuss, 2022). Atropine consists of a tropine organic base and a tropic aromatic acid with an organic ester. These nonselective and antimuscarinic compounds have a greater affinity to bind with five muscarinic acetylcholine receptors viz., M1, M2, M3, M4 and M5 (Korczynska et al., 2018). The ester is mainly responsible for the activity of atropine, whereas tropine base and tropic acid are inactive to execute their function. Low dose-dependent administration of the drug atropine is used by intravenous (IV), subcutaneous, intramuscular, or endotracheal (ET) methods. Several drugs are present in the market derived from atropine by different commercial medicine industries. As discussed earlier, this compound treats eyesight and cardiovascular problems, and medicine has been developed based on its function. Such as atropine ophthalmic under the brand name of Atropisol used to treat pupillary dilation, refraction, assessment, and Uveitis. Another drug atropine systemic under the brand name of AtroPen is used in treatment of anticholinesterase poisoning, AV heart block, bradyarrhythmia, and Organophosphate Poisoning. Furthermore, atropine/difenoxin systemic is a drug named Motofen, often used to treat diseases such as diarrhea (Jalilzadeh-Amin and Maham, 2015). With this extended spectrum of activity, this compound is used as a commercial drug and associated with other drugs to treat different deadly diseases.

### 5.2 Hyoscyamine

Hyoscyamine is another member of tropane alkaloids class of compounds. The compounds are used in medicine as an anticholinergic drug. The plants belonging to the family Solanaceae such as *Anisodus* spp., *Atropa belladonna*, *Datura* spp., *Duboisia* spp., *Hyoscyamus* spp., *Przewalskia tangutica*, *Scopolia japonica* produces this compound in their root as well as in leaf (Nabeshima et al., 1986; Sauerwein and Shimomura, 1991; Dupraz et al., 1993; Uchida et al., 1993; Ur Rahman et al., 2006; Li et al., 2008; Lan and Quan, 2010; Qin et al., 2014; Zeynali et al., 2016; Ullrich et al., 2017; Khezerluo et al., 2018). Elevated production of this compounds is also reported in the presence of different chemical substrate such as jasmonic acid in *Datura stramonium* (Amdoun et al., 2018). The structure of hyoscyamine is complex and costly to synthesize chemically. So, the production of this bioactive compound is mainly plant-based. Obtaining the plants throughout the year, is difficult due to their availability in natural environments in a particular time and area. Alternatively, it was reported that genetically transformed roots i.e., hairy roots of *Hyoscyamus niger* are the most prominent site for hyoscyamine production in enhanced rate (Jouhikainen et al., 1999). Hyoscyamine is mainly used to relieve stomach spasm, muscle spasms, arterial spasm, ulcer pain and other blood circulation diseases (Dehghan et al., 2017). Hyoscyamine has also been reported to effectively treat hypervagotonia causing advanced AV block, as a muscarinic receptor blocker, and it is an attractive treatment option for patients with hypervagotonia, as it can substitute permanent pacemaker implant (Mai and Kusumoto, 2022). According to previous reports, hyoscyamine is produced from hyoscyamine aldehyde through enzymatic reaction lead by hyoscyamine dehydrogenase. The enzyme is also able to oxidize hyoscyamine to form hyoscyamine aldehyde. Overexpression of the enzyme hyoscyamine dehydrogenase is also important for enhanced and elevated hyoscyamine production (Qiu et al., 2021). Hence, hyoscyamine’s pharmaceutical need must be met by using hyoscyamine dehydrogenase as an alternative approach for enhanced production. Another enzyme Hyoscyamine 6β-hydroxylase (H6H) is committed to execute the last step of biochemical pathway of hyoscyamine which ultimately converts it into scopolamine (Kai et al., 2012b; Cardillo et al., 2013).

### 5.3 Scopolamine

Scopolamine is one more tropane alkaloid with significant medicinal properties (Ogunsuyi et al., 2018). Scopolamine has limited side effects and more potent pharmacological effects compared to the tropane alkaloid hyoscyamine (Kai et al., 2018). Hence, the demand for scopolamine is more, with a high market value. Memory loss is a devastating human disorder due to diseases like amnesia, dementia, schizophrenia, epilepsy and depression and Alzheimer’s disease. A progressive mode of neurodegeneration can result in this type of disease. Scopolamine has been used in perspective to cure this neurogenerative disorder by readily inhibiting parasympathetic nerve block. Therefore, scopolamine is also used to improve circulation, detoxification, Parkinson’s disease, anesthesia, analgesia, anti-motion sickness, pesticide poisoning, etc. Furthermore, scopolamine is used to treat chronic obstructive pulmonary disease (COPD) by FDA (Koumis and Samuel, 2005). Therefore, the compound is essential and commercial production is very much needed. An enormous amount of scopolamine synthesis is reported several times in plants’ hairy roots of *Atropa baetica*, *Atropa belladonna*, *Datura metel*, *Duboisia leichhardtii*, *Hyoscyamus muticus*, *Hyoscyamus niger* and *Hyoscyamus reticulatus* (Kamada et al., 1986; Jaziri et al., 1988; Mano et al., 1989; Ionkova, 1992; Zárate, 1999; Moyano et al., 2003; Zárate et al., 2006; Zhang et al., 2007; Zolala et al., 2007; Singh et al., 2018; Moradi et al., 2020; Shameh et al., 2021). Transgenic roots are reported for higher rate of production of scopolamine (Singh et al., 2018). The structures of scopolamine have one asymmetric carbon atom or chiral center localized in the tropic acid component. The pKa value of this compound is 7.6, with a weak essential character and good lipid solubility with a partition coefficient of 1.2 (Renner et al., 2005). Production of the tropane alkaloids depends on a few rate-limiting enzymes and gene expression regarding these enzymes. These genes, such as *PMT*, *TRI*, *TRII*, *CYP80F1*, and *H6H* are related to the production of tropane alkaloids. Overexpression of these genes through metabolic engineering, and biotransformation of hyoscyamine to scopolamine, has been considered as great approaches for production enhancement (Palazón et al., 2008). The biosynthesis of tropane alkaloids largely depends on the putrescine *N*-*methyltransferase* gene (*PMT*), which belongs to the N-methylation transferase enzyme family. The PMT gene is responsible for the methylation of putrescine for tropane alkaloids biosynthesis (Kohnen-Johannsen and Kayser, 2019). Overexpression of this gene results in the high yield of scopolamine in plant’s hairy roots of various Solanaceous plants (Hibi et al., 1994). The plant *Anisodus acutangulus* was reported for two *PMT* genes such as *AaPMT1* and *AaPMT2*, which are mainly expressed in roots and weakly expressed in stem and leaf. Therefore, the production of tropane alkaloids begins in the respective plant. *Tropinone reductase I* (*TRI*) and *tropinone reductase II* (*TRII*) are both tropane alkaloids-producing genes that are reported for the elevated synthesis of particular alkaloids in Solanaceous plants (Hashimoto et al., 1992). The *hyoscyamine 6β-hydroxylase* (*H6H*) gene is reported for synthesizing scopolamine by converting hyoscyamine derived from tropine. H6H is a bifunctional enzyme with a two-step completion mechanism of scopolamine biosynthesis. In the first step, the enzyme catalyzes the reaction of hydroxylation of hyoscyamine with the formation of 6β-hydroxy hyoscyamine and then the epoxidation of 6-hydroxy hyoscyamine to scopolamine (Kai et al., 2012b; Xia et al., 2016). Cloning and genetic engineering of the H6H gene have been done to increase the production of scopolamine through *Agrobacterium*-mediated transformation in *A. acutangulus*. The elicitation for enhanced production of scopolamine is achieved by treating the recombinant strain with salicylic acid (SA), methyl jasmonate (MJ), and acetylsalicylic acid (ASA). The best increase in scopolamine production was seen in methyl jasmonate treatment, followed by acetylsalicylic acid dissolved in ethanol (El Jaber-Vazdekis et al., 2008). To achieve the highest production of hairy roots and bioactive compounds, the bioreactor-mediated production of secondary metabolites was also reported in these elicitors’ presence. About 146% of scopolamine production in roots in the hybrid bubble column/spray bioreactor has also been reported (Jaremicz et al., 2014).

### 5.4 Solasodine

Solasodine is a vital steroidal alkaloid in Solanaceous plants such as potatoes and tomatoes with an inordinate bio-significance. The compound is pharmaceutically viable and used for medication in different aspects. According to the previous literature, solasodine has anticancer, diuretic, cardioprotective, anti-androgenic, antibacterial, antifungal, anti-spermatogenetic, immunomodulatory activities, and also has effects on the central nervous system (Kumar et al., 2019). Solasodine O-(diethyl phosphate) and N-acetyltetrahydrosolasodine are the derivatives of solasodine. Solasodine is a spiroketal alkaloid sapogenin consisting of C27 cholestane, and a 1-5 carbohydrate side chain is hung at the 3-OH region of the aglycone. Several studies have been conducted for anticancer activities of solasodine and reported to be very effective in curing cancer. As per previous studies, different types of cancer, such as sarcoma, myeloma and leukemia, have been treated by solasodine till date. Molecular investigation corresponding to the mode of action of solasodine revealed that rhamnose in the solasodine glycosides binds with the tumor cell and inhibits them. Zycure (solasodine glycosides) was demonstrated as an effective drug at a 0.005% mixture, exhibiting 66% and 78% curable percentage on 56 days and 1-year follow-up, respectively (Punjabi et al., 2008). Apoptosis induction by solasodine was also reported due to their carbohydrate moiety (C3 side chain) and conformation at C-5 and C-25. Furthermore, cell cycle arrest, cell toxicity and uncontrolled cell proliferation inhibition were also been reported several times by different researchers (Akter et al., 2015). Solasodine has free radical scavenging properties. Therefore, the compound has neuroprotective activities and protects the brain’s coronal region, according to histopathological studies (Al-Rehaily et al., 2013). The neuroprotective activity was assayed against ischemia in rats, and the enhancement of GSH, CAT and suppression of LPO and NO was observed. Solasodine has also been reported for its anti-inflammatory activities by reducing tetradecanoyl-phorbol 13-acetate-mediated inflammation in the ear. Despite these, solasodine is well-known for its high antimicrobial, antiviral, anti-aging, and immune-protective activities (Patel et al., 2013). Production of solasodine in hairy roots of solanaceous plants including *Physalis minima*, *Solanum aviculare*, *Solanum erianthum*, *Solanum mauritianum*, *Solanum virginianum*, and *Solanum trilobatum* has been reported and productions have been further enhanced (Subroto and Doran, 1994; Drewes and Van Staden, 1995; Yu et al., 1996; Kittipongpatana et al., 1998; Argôlo et al., 2000; Putalun et al., 2004; Pawar Pankaj et al., 2008; Shilpha et al., 2015; Sarkar et al., 2020).

### 5.5 Withaferin A

Withaferin A is a C28 steroidal lactone with four cycloalkane rings comprised of three cyclohexane and one cyclopentane ring. The lactone part consists of five carbon atoms with a single oxygen atom. The activity of withaferin A is majorly dependent on the lactone ring. One ring is unsaturated and contains ketone, whereas the other ring is an epoxide responsible for cytotoxicity. Withaferin A is found largely in different plant species belonging to the family Solanaceae. The presence of this compound in the plants makes them biologically active in treating different diseases such as solid tumors, rheumatoid arthritis and endometriosis, also known as angiogenesis (Folkman et al., 1971; Colville-Nash and Scott, 1992; Reynolds et al., 1992). Withaferin A reported for its anticancer activities against different types of cancer. According to previous reports, sarcoma, prostate, gynaecological, melanoma, thyroid, gastrointestinal, and other cancer was curable by using this chemical compound. Withaferin A can cause regression of tumor cells by decreasing the angiogenesis marker CD31, increasing the expression of BAX, and activating caspase 3 through inhibition of NF- κβ signalling pathway (Yang et al., 2007). The biochemical basis of anticancer activities of withaferin A is dependent on some aspects such as suppression of inflammatory pathways (Min et al., 2011; Lee et al., 2012; SoRelle et al., 2013), angiogenesis (Reynolds et al., 1992), reinforcement of detoxification system (Manoharan et al., 2009; Panjamurthy et al., 2009), induction of apoptosis by prevention of cell proliferation and tumor invasion, prevention of cytokine storm, cell metabolism alteration and eradication of cancer stem cells (Behl et al., 2020). Including anticancer activities, withaferin A has Anti-Inflammatory activities, achieved by the elevation of various pro-inflammatory mediators like cytokines. Nitric oxide and prostaglandin regulation is another pathway to regulate inflammation in the system by elevated expression of COX-2 and iNOS. As a result, prevention and promotion of cancer progression are inhibited (Kundu and Surh, 2008). There are more different pathways to prevent inflammation by withaferin A reported till date (Lee et al., 2012; Ku et al., 2014). Another important activity of Withaferin A reported was antiproliferative activity achieved by G2/M phase cell cycle arrest (Munagala et al., 2011). Reduction of expression of Bcl-2 mediated inhibition of phosphatidyl inositol-3 kinase (PI3K)/Akt signalling can also reduce cancer cells by arresting them G0/G1 phase (Cai et al., 2014). Anti-diabetic activities of withaferin A have been reported several times in previous literature. Type I diabetes meletus solely depends on insulin treatment as the therapeutic strategy. As an alternative therapy, withaferin A can be used due to its potential to alter lipid and glucose metabolism pathways. Obesity is an evident parameter regarding diabetes, which is caused due to inflammatory mediators such as TNF-α, IL6, and resistin. These mediators can negatively regulate insulin signalling by phosphorylating insulin receptor substrates 1 (IRS-1). This negative signalling can be controlled by withaferin A treatment by downregulating insulin signalling gene expression. According to previous research, Insulin signalling gene expression is alternatively dependent on the anti-inflammatory response of a compound. As described in the previous section, Withaferin A is potent towards negative regulation of inflammatory response and is used as medicine for anti-inflammatory activities. So, this chemical substance can be used as an alternative drug resource in treating diabetes mellitus (Khalilpourfarshbafi et al., 2019). Other mechanisms of action were reported to control diabetes, such as caspase 3 upregulation, apoptotic body formation, DNA fragmentation, and cytoplasmic-nuclear condensation (Riboulet-Chavey et al., 2008). Neuro-degradation is a devastating human disorder and causes death due to immature neurotransmitter degradation. Dopamine (DA) and homo vanillic acid (HVA) are the neuroprotective compounds in the human system. The elevation of these compounds can be achieved by treating Withaferin A at 50 mg/kg body weight (Banu et al., 2019). Brain injury is a deadly concern worldwide which can be treated by the treatment of withaferin A. The compound has an activity to reduce histological alteration in tissues after injury by elevating neurobehavioral functions. The compound can also reduce apoptosis in endothelial cells and disrupt the blood-brain barrier (Zhou et al., 2020). Withaferin A is also known for its cardioprotective activity. Myocardial infection is a serious health concern and causes the death of humans every year worldwide. Treatment of this issue can be achieved by using withaferin A at a low concentration of dose of 1 mg/kg/mice. Low concentration of the compounds leads to the mitochondrial apoptotic pathway by upregulating the Bcl-2 protein. Withaferin A can reduce the mitochondrial apoptotic pathway by acting on the AMPK pathway by regulating the Bcl-2/Bax ratio. Therefore, it can treat cardiovascular disorders as a drug (Guo et al., 2019). Withaferin A was reported in the treatment of the recent pandemic Covid 19 also by some researchers. Covid 19 is a deadly viral infection that causes different immune reactions by releasing pro-inflammatory cytokines known as a cytokine storm. Withaferin A was reported as an anti-inflammatory substance, as discussed in the earlier section of this study, and can decrease the release of different pro-inflammatory cytokines such as IL-6, TNFα, IL-8, IL-18. Therefore, it can control the infection caused by covid 19 (Straughn and Kakar, 2019). Furthermore, Anti Hepatitis and osteoporosis activity of withaferin A have also been reported by several researchers recently (Farrell and Larter, 2006; Khedgikar et al., 2013; Michelotti et al., 2013; Tit et al., 2018). Enhancement in production of withaferin A in hairy roots of *Withania coagulans*, *Withania somnifera*, *Physalis minima* species has been reported (Saravanakumar et al., 2012; Halder and Ghosh, 2022; Karami et al., 2023).

### 5.6 Other important secondary metabolites synthesized by hairy roots of solanaceous plants

Besides the above mentioned compounds, other metabolites like 4β-hydroxywithanolide E, Aculeatiside A and B, Anisodine, Physagulins, Physalins, Kukoamine A, and Nicotine are found in this family, which are considered as important bioactive compounds. However, limited studies have been conducted on the hairy root cultures of these plants.

#### 5.6.1 4β-hydroxywithanolide E

This is a naturally occurring bioactive compound present in most of the edible plants and first reported in golden berry (*Physalis peruviana*). It is a derivative of withanolide with a 17α-oriented side chain (Sakurai et al., 1976). The compound has a high potential towards disease control including oral cancer, lung cancer, colorectal cancer adipogenesis inhibition through apoptosis, cell cycle arrest, and through modulation of mitotic clonal expansion respectively (Chiu et al., 2013; Ye et al., 2019; Hsieh et al., 2021; Kumagai et al., 2021). The compound also has been reported earlier due to attenuation of NF-κB signalling resulting in anti-tumor activity against various cancer cells (Takimoto et al., 2014). According to Yang et al. (2020) chronic obstructive pulmonary disease (COPD) also can be controlled by treating 4β-hydroxywithanolide E. Due to this type of diverse pharmacological significance, enhanced production of this compound is necessary to combat the disease described above. Therefore, enhanced production has been carried out by transforming the roots of *Physalis peruviana* L. by using *Agrobacterium rhizogenes* to achieve the emerging need of 4β-hydroxywithanolide E (Piñeros-Castro et al., 2009).

#### 5.6.2 Aculeatiside A and B

Steroidal glycosides are known to be biologically active and used in treatment of different diseases. Aculeatiside A and B are two naturally occurring steroidal glycosides found in solanaceous plants (Saijo et al., 1983). Both compounds are known for their pharmacological application and significance. Aculeatiside A is reported as cytotoxic substance and have the ability to eliminate hepatocellular carcinoma cells by inhibit the growth of HepG-2 cells significantly by induction of apoptosis (Nafady and Mohafez, 2011). According to Fujiwara et al. (2018) aculeatiside A can induced CD169-positive phenotype in human monocyte-derived macrophages. In addition, by enhancing the content of IL-1β, IL12, and CD169, aculeatiside A enhance anti-tumor immune responses also.

#### 5.6.3 Anisodine

This is an important alkaloid naturally found in *Solanaceae* spp., with a molecular structure of tropine-6-hydroxy-3-depinate. Anisodine, atropine and scopolamine are commonly known for their activity towards blocking M-choline receptor. Implication of anisodine has been developed the field of medicine and basic research till date. The compound is often used to treat septic shock, blood circulation obstruction, and retinal protection (Zhang et al., 2023b). Anisodine can’t cross the blood–brain barrier due the presence of β-oriented hydroxyl group on them. As a result, low central excitation of nervous system takes place which resulted in less amount of pharmacological impact.

#### 5.6.4 Physagulins

Another group of alkaloids found in solanaceous plants (like *Physalis angulata* L.) are physagulin A, physagulin C, and physagulin H reported for their potential application towards anti-inflammatory activity through NF-κB inhibition (Wang et al., 2021). Therefore, these group of compounds are used as anti-inflammatory drug to treat acute inflammations. *A. rhizogenes-mediated* gene transformation in *Physalis angulata* has been reported previously by Zhang et al. (2018) for enhanced production of physagulin A, B, and C. This is an alternative way of enhanced production of these compounds to reach the demand during treatment.

#### 5.6.5 Kukoamine A

Another pharmacologically important anti-inflammatory spermine alkaloid is ‘kukoamine A’, found in the root bark of Solanaceous species. The compound is well known for its varied medicinal significance including anti-inflammatory, antioxidant, cytoprotective, control of blood pressure, and inhibition of human glioblastoma cell growth and migration through apoptosis induction (Wang et al., 2016; Li et al., 2018; Wang et al., 2020; Butts et al., 2022). Due to these bioactivities kukoamine A is used in medicine for the treatment of respective disease. Therefore, the requirement of this compound is naturally high. Hairy root production by transforming the plant *Lycium ruthenium* with the help of *A. rhizogenes* has been reported for enhanced production of kukoamine A (Chahel et al., 2019).

#### 5.6.6 Physalins

This steroidal group of compounds are found in the plants belonging to the genus *Physalis*. Physalin A, B, C, D, F, G, H, L, and P is the predominant compounds among all the physalin group of compounds. All the compounds described above are reported for their important bio-activities including anti-cancer, anti-inflammatory, Immunoregulatory, antimicrobial, trypanocidal, leishmanicidal, antinociceptive, and antidiabetic activities (Wu et al., 2021). A large quantity of these compound is required for the synthesis of drug and to treat the diseases. The large amount of compound can be synthesized by production of hairy root in *Physalis angulata* through genetic transformation has been reported by Zhang et al. (2018). According to the previous reports, another two important pharmacokinetic compounds hygrine and cuscohygrine found in the transformed hairy roots of *Nicandra physalodes and Hyoscyamus niger* respectively (Parr, 1992; Jaremicz et al., 2014). Both compounds are reported as good markers to distinguish between chewing coca leaves and cocaine abuse, and also used in drug testing and forensic cases (Rubio et al., 2013). Therefore, enhanced amount of these compounds can be obtained by production of hairy roots through genetic transformation.

#### 5.6.7 Nicotine

This is a naturally occurring alkaloid consisting of a pyridine ring, a pyrrolidine ring, and a methyl group. It is found primarily in tobacco plants (*Nicotiana tabacum*). The structure of nicotine allows it to interact with nicotinic acetylcholine receptors (nAChRs) leading to the release of neurotransmitters such as dopamine, norepinephrine, and serotonin, which contribute to various effects on mood, cognition, and reward pathways. Beside the addictive properties, it is pharmacologically important due to its effects on the central nervous system. It has been investigated for its cognitive-enhancing effects, potential neuroprotective properties, and its role in certain neurological disorders and nicotine replacement therapy. Hairy root cultures of *Nicotiana tabacum* have been extensively studied to enhanced nicotine production optimizing growth conditions, nutrient supplementation, and genetic engineering (Zhao et al., 2013; Kumar and Mitra, 2017).

## 6 Biotechnological applications of solanaceous hairy roots other than secondary metabolite production

Besides secondary metabolite production, hairy roots are being considered as an alternative system for the biosynthesis of different plant-derived valuable compounds. Hairy roots of different solanaceous plants have emerged as valuable tools for producing recombinant protein, antigen and monoclonal antibody (Roychowdhury et al., 2012). Successful production and secretion of recombinant protein thaumatin into the tobacco hairy root culture medium was achieved by Pham et al. (2012). Hairy root culture system from *Nicotiana tabacum* was also used for the production of recombinant human EPO (rhEPO), which is regularly produced in mammalian cells (Gurusamy et al., 2017). *N. tabacum* hairy roots are also utilized for the production of recombinant LSC protein (Fallah et al., 2018). Gene transfer through an improved viral vector is the most significant technique to introduce a gene into the plant, which boosts production of antigens for further vaccine development. Optimization of PD-FcY veterinary antigen secretion from *N. benthamiana* hairy roots and purification from the culture medium was done by Rage et al. (2020). B-subunit of the heat-labile toxin (LTB) of *Escherichia coli* is the causative protein for causing enterotoxigenic disease in humans. According to De Guzman et al. (2011) enhanced production of this antigen has been achieved in *N. tabacum*, *Solanum lycopersicum* and *Petunia parodii* plants by introducing the LTB gene under the control of CaMV35S promoter followed by transformation. Many species of Solanaceae family are primarily known as edible crops. So, the delivery of the pharmaceutical proteins through consumption of food can be done in the safest and cost-effective ways. Hepatitis B virus is the most dominant viral infection with high death rate. Production of HBsAg surface antigen present on the outer surface of the virus can help to develop vaccine against HBV. Hairy root mediated enhanced production of HBsAg has been reported by Kumar et al. (2006) in potato. Such type of antigen-mediated vaccine production has been reported previously for other diseases also (Rigano et al., 2013). Hairy root cultures were used to express a harmless form of the HPV type 16 E7 protein (E7*) fused to SAPKQ, a noncytotoxic form of the saporin protein from *Saponaria officinalis* and E7*-SAPKQ candidate vaccine were obtained upon infection of leaf explants of *Solanum lycopersicum* using a recombinant plant expression vector (Massa et al., 2019). Novel hydroxyproline (Hyp)-O-glycosylated peptides (HypGPs) have been engineered into tobacco hairy roots to boost the extracellular secretion of fused proteins by Zhang et al. (2019). Enhanced production of monoclonal antibody IgG1, effective against different viral and bacterial infection, has also been reported in hairy roots of tobacco plants by Wongsamuth and Doran (1997). Additionally, *Nicotiana tabacum* hairy roots are reported for enhanced production of 14D9 antibody with an improved yield of 20.82 µg/ml (Martínez et al., 2005). According to Häkkinen et al. (2014) monoclonal antibody M12 production has been achieved in hairy roots of *Nicotiana*. Several functional monoclonal antibodies and the tumor-targeting monoclonal antibody H10, were produced in hairy roots of *Nicotiana tabacum* and *N. benthamiana* (Lonoce et al., 2016). Production of anti-fungal scFvFc 2G8 antibody against the most potent fungal pathogen *Candida albicans*, has been achieved in hairy roots of *Nicotiana benthamiana* (Catellani et al., 2020).

Hairy roots are used to phytoremediate persistent organic pollutants (POPs) from the agriculture fields as they resemble the normal roots of the mother plants physiologically (Majumder and Jha, 2012; Majumder et al., 2018; Nanasato and Tabei, 2018). Hairy roots of the plants in the Solanaceae family, have been used in phytoremediation due to their ability to accumulate and detoxify various pollutants. *Datura inoxia* has been used in phytoremediation studies for heavy metals, such as cadmium and lead (Waoo, 2016). *Datura stramonium* has been investigated for its potential in phytoremediation of pollutants like petroleum hydrocarbons, polycyclic aromatic hydrocarbons (PAHs), and heavy metals (Padmavathiamma et al., 2014). *Solanum lycopersicum* hairy roots, have shown potential for the remediation of organic contaminants and heavy metals. Hairy root system of *Capsicum annuum* plants have also been investigated for phytoremediation studies, particularly for heavy metal uptake. Phytoremediation of phenol by hairy roots of *Solanum aviculare* has been reported earlier by De Araujo et al. (2006). According to them, 98.6% of the phenol in the medium has been remediated by *Solanum aviculare* within 72 hrs. Another report suggests that the *Solanum nigrum* mediated hairy roots are able to metabolize PCBs at a higher rate in a short time (Kučerová et al., 2000 and Rezek et al., 2007). Hence, enhanced production of hairy roots by genetic transformation can help to remediate such compounds in future.

## 7 Conclusion and prospects

Production of bioactive compounds, intended for industrial use, needs guarantee of a high-value quality product, which may not be achieved through cultivated or natural plants. In this situation, *in vitro* transformed cultures ensure an uninterrupted supply of the safe and superior quality product at a lower cost. Compared to other cultures, highly differentiated hairy root cultures appear to be potentially better system for production of valuable secondary metabolites. This study explores various strategies for optimizing secondary metabolite production in solanaceous plants using hairy root cultures, including genetic engineering, elicitation with biotic and abiotic elicitors, optimization of culture conditions, and scale-up strategies. The application of these techniques has demonstrated promising results in enhancing the yield and quality of secondary metabolites. In addition to the external stimuli, secondary metabolic pathways have also been modified for enhanced metabolite production through metabolic engineering, such as overexpression of biosynthetic genes and transcription factors, and suppression of catabolic or competing pathway genes. Genetic and biochemical stability of the hairy roots, as well as its high productivity offers an effective platform for further studies on the biosynthetic pathways of phytochemicals of solanaceous plants.

The production of secondary metabolites through hairy root culture also presents ecological and economic benefits. It reduces the pressure on traditional plant sources, such as endangered or slow-growing species, by providing an alternative sustainable method for obtaining valuable compounds. Additionally, the scalability of hairy root cultures warrants commercialization and widespread utilization of these valuable compounds. The optimization of culture conditions, bioreactor design, and process engineering can facilitate a consistent supply of high-quality metabolites.

Furthermore, hairy root culture holds immense potential as a tool for studying CRISPR/Cas9 (Cas9)-mediated gene editing in a wide range of plants. Very recently, genome editing techniques like meganucleases, ODM (Oligonucleotide-directed mutagenesis), ZFNs (Zinc finger nucleases), TALENs (transcription activator-like effector nucleases) and CRISPR/Cas9 (clustered regularly interspaced short palindromic repeats) have been widely used for genome modifications. For medicinal plants, CRISPR/Cas9 technique has proved to be a valuable method for modifying metabolic pathways and developing plants with optimized secondary metabolite production. Future work should include CRISPR/Cas9-directed genome editing and more genetic manipulations, coupled with efficient metabolite extractions to allow full exploitation of hairy roots of these plants as a production platform of valuable secondary metabolites.

With the introduction of next-generation sequencing (NGS) methods, hairy root cultures have emerged as valuable tools in analyzing and identifying the appropriate genes/enzymes of the biosynthetic pathways for secondary metabolites production. They also have promising future in allowing RNA-Seq, to investigate even low-abundance transcripts with high accuracy and precision. This method is used for depicting the expression status of the genes in plants with or without available reference genome sequence. The genetic biosynthetic pathways of the plants can also be traced using high-throughput technologies such as genomics, proteomics, metabolomics, and transcriptomics. Several studies aim to characterize biosynthetic pathways of phytochemicals, and efforts are on identifying genes, proteins, and metabolomes associated with a particular metabolite in hairy root culture system. All this has led to an emergence of an entirely new discipline known as phytochemical genomics, which involves the integration of multi-omics approaches.

Concludingly, among plant-based expression formats, hairy root culture is a robust platform act as a plant-based bioreactors. This establishes the suitability of hairy root cultures as possible bio-factories of future decades to produce tailor-made compounds.

## Author contributions

AC, DB, and SM wrote the main manuscript and prepared the table and figure. SM and BG critically reviewed the manuscript. All authors contributed to the article and approved the submitted version.
