# Supplementary material for: Hairy root culture: a potent method for improved secondary metabolite production of Solanaceous plants
Source: Front Plant Sci. 2023 Sep 4;14:1197555. doi: 10.3389/fpls.2023.1197555 (PMC10507345; doi:10.3389/fpls.2023.1197555)
Supplement: Supplementary file 1 [file Table_1.docx]

**Table 1. Secondary metabolite production enhancement strategies in hairy roots of Solanaceous species**

| **Sl. No.** | **Secondary metabolite** | **Plant** | **Bacterial strain** | **Vector/ Genetic**  **construct** | **Extraction**  **solvent** | **Strategy used for production enhancement** | **Changes in secondary metabolite profile from control roots** | **References** |
| --- | --- | --- | --- | --- | --- | --- | --- | --- |
|  | 4β-hydroxywithanolide E | *Physalis peruviana* L. | *Agrobacterium rhizogenes* strain C106 | **—^⁕^** | Water | **—** | Increased production of 4β-hydroxywithanolide E | (Piñeros-Castro et al., 2009) |
|  | Aculeatiside A | *Solanum aculeatissimum* Jacq. | *A. rhizogenes* strains 15834 | **—** | Ethanol | **—** | Production of Aculeatiside A | (Ikenaga et al., 1995) |
|  | Aculeatiside B | *Solanum aculeatissimum* Jacq. | *A. rhizogenes* strains 15834 | **—** | Ethanol | **—** | Production of Aculeatiside B | (Ikenaga et al., 1995) |
|  | Anabasine | *Nicotiana glauca* Graham | *A. rhizogenes* | **—** | 0.01 N H_2_SO_4_ | **—** | Double production of anabasine | (Fecker et al., 1992) |
|  |  | *Nicotiana rustica* L. | *A. rhizogenes* strain  LBA9402 | **—** | 2% NH_4_OH, chloroform | **—** | Increased production of anabasine | (Walton et al., 1988) |
|  | Anatabine | *Nicotiana rustica* L. | *A. rhizogenes* strain  LBA9402 | **—** | 2% NH_4_OH, chloroform | **—** | Production of anatabine | (Hamill et al., 1986) |
|  |  |  | *A. rhizogenes* strain  LBA9402 | **—** | 2% NH_4_OH, chloroform | **—** | Increased production of anatabine | (Robins et al., 1987) |
|  |  |  | *A. rhizogenes* strain  LBA9402 | **—** | 2% NH_4_OH, chloroform | **—** | Production of anatabine | (Benson and Hamill, 1991) |
|  | Anisodamine | *Brugmansia* × *candida* Pers. | *A. rhizogenes* strain  LBA9402 | **—** | 0.2M H_2_SO_4_, chloroform | Hairy root culture in modified stirred-tank reactor | Higher amount (10.05 ± 0.76 mg/g) productions of anisodamine | (Cardillo et al., 2010) |
|  |  | *Hyoscyamus niger* L. | *A. rhizogenes* strain LBA1334 | **—** | Chloroform:methanol: ammonium hydroxide (15:5:1) | Hairy root culture using bubble-column  bioreactor and a hybrid bubble-column/spray bioreactor | Increased anisodamine production | (Jaremicz et al., 2013) |
|  | Anisodine | *Anisodus acutangulus* C.Y.Wu & C.Chen | *A. tumefaciens* strain C58C1  containing *A. rhizogenes* Ri plasmid | *p*CAMBIA1304^+^ containing *AaTRI* and *AaH6H* | Chloroform:methanol: ammonium hydroxide (15:5:5) | Metabolic engineering through overexpression of *TRI* and *H6H* gene | 18.57 fold increased production of anisodine | (Kai et al., 2012) |
|  | Atropine | *Anisodus luridus* Link | *A. rhizogenes*, strain ATCC 15834 | **—** | **—** | **—** | Production of atropine | (Jovanović et al., 1991) |
|  |  | *Atropa belladonna* L. | *A. rhizogenes* strain ATCC 15834 | *p*Ri 15834 | Chloroform:methanol: ammonium hydroxide (15:5:1) | **—** | Higher productions of atropine | (Kamada et al., 1986) |
|  |  | *Datura metel* L. | *A. rhizogenes* A4 | **—** | Ethanol containing 2% H_2_SO_4_ | Elicitation using nanosilver | Enhanced production of atropine | (Shakeran et al., 2015) |
|  |  | *Hyoscyamus albus* L. | *A. rhizogenes* strains,  LBA9402 and A4 | **—** | Methanol, 2% HCl | **—** | 3.5 fold enhanced atropine production | (Zehra et al., 1998) |
|  | Curcumin | *Atropa belladonna* L. | *A. rhizogenes* strain ATCC 15834 | **—** | Methanol | Hairy root culture in modified stirred-tank reactor | 2.3 fold enhanced curcumin production | (Singh et al., 2021) |
|  | Cuscohygrine | *Hyoscyamus niger* L. | *A. rhizogenes* strain LBA1334 | **—** | Chloroform:methanol: ammonium hydroxide (15:5:1) | Hairy root culture using bubble-column  bioreactor and a hybrid bubble-column/spray bioreactor | Increased cuscohygrine production | (Jaremicz et al., 2013) |
|  | Hygrine | *Nicandra physalodes* (L.) Gaertn. | *A. rhizogenes* strain  LBA9402 | **—** | Alkaline chloroform | **—** | Production of hygrine | (Parr, 1992) |
|  | Hyoscyamine | *Anisodus acutangulus* C.Y.Wu & C.Chen | *A. tumefaciens*  strain C58C1 | *p*RiA4 | Chloroform:methanol: ammonium hydroxide (15:5:5) | **—** | 1.5-fold higher production of hyoscyamine | (Li et al., 2008) |
|  |  |  | *A. tumefaciens* strain C58C1 | *p*RiA4 and  *p*CAMBIA1304^+^ carrying *AaTRI* | Chloroform:methanol: ammonium hydroxide (15:5:5) | *AaTRII* gene transformed hairy root culture | 1.87-fold higher  level production of hyoscyamine | (Kai et al.. 2009) |
|  |  | *Anisodus luridus* Link | *A. tumefaciens* strain C58C1 | *p*RiA4 | Ethanol:28% ammonium hydroxide (19:1) | **—** | Production of hyoscyamine | (Qin et al., 2014) |
|  |  | *Atropa belladonna* L. | *A. tumefaciens* strain  C58C1 | *p*RiA4, *p*XI containing *pmt* and *h6h* | Ethanol:28 % ammonium hydroxide (19:1) | Metabolic engineering through overexpression of *pmt* and *h6h* gene | 11 fold enhancements in hyoscyamine production | (Yang et al., 2011) |
|  |  |  | *Agrobacterium rhizogenes* strain R15834 | **—** | 95% ethanol, chloroform | Elicitation by methyl jasmonate | Increased production of hyoscyamine | (Moradi et al., 2020) |
|  |  | *Brugmansia* × *candida* Pers. | *A. rhizogenes* strain  LBA9402 | **—** | 0.2% H_2_SO_4_, chloroform | Elicitation by hemicellulose, homogenized *Brugmansia candida* roots and CuSO_4_ | Enhanced production of alkaloids including hyoscyamine | (Pitta-Alvarez and Giulietti, 1998) |
|  |  |  | *A. rhizogenes* strain  LBA9402 | **—** | 0.2% H_2_SO_4_, chloroform | **—** | Elicited productions of hyoscyamine | (Pitta-Alvarez et al., 2000) |
|  |  |  | *A. rhizogenes* strain  LBA9402 | **—** | 0.2% H_2_SO_4_, chloroform | **—** | Superior productions of hyoscyamine | (Pitta-Alvarez et al., 2003) |
|  |  | *Datura metel* L. | *Escherichia coli* strain DH5a and *Agrobacterium tumefaciens*  strain C58C1 | *p*MBI and *p*RiA4 | Ethanol: 28% ammonium hydroxide (9:1) | Metabolic engineering through overexpression of *pmt* gene | Enhanced production of hyoscyamine | (Moyano et al., 2003) |
|  |  | *Datura quercifolia* Kunth | *A. rhizogenes* strain  LBA9402 | *p*Ri1855 | Chloroform:methanol: ammonium hydroxide (15:5:1) | **—** | Production of hyoscyamine | (Dupraz et al., 1993) |
|  |  | *Datura stramonium* L. | *A. rhizogenes* strains A4 | **—** | Hexane | **—** | Production of hyoscyamine | (Amdoun et al., 2009) |
|  |  |  | *A. rhizogenes* strains 15834 | **—** | Methanol, 3% H_2_SO_4_ | **—** | Production of hyoscyamine | (Pavlov et al., 2009) |
|  |  |  | *A. rhizogenes* strains A4 | **—** | Hexane | Jasmonic acid elicitaion | 290% increase in hyoscyamine production | (Amdoun et al., 2018) |
|  |  | *Duboisia leichhardtii* F.Muell. | *Agrobacterium rhizogenes* strain ATCC 15834 and LBA9402 | *H6H* and *HCHL* | Chloroform:methanol: ammonium hydroxide (15:5:1) | **—** | Production of hyoscyamine | (ur Rahman et al., 2006) |
|  |  | *Duboisia myoporoides* R.Br. | *A. rhizogenes* strain  HRI | **—** | **—** | **—** | Lower production of hyoscyamine | (Deno et al., 1987) |
|  |  | *Hyoscyamus albus* L. | *A. rhizogenes* strain MAFF 03-01724 | **—** | Chloroform:methanol: ammonium hydroxide (15:5:1) | **—** | Production of hyoscyamine | (Sauerwein and Shimomura, 1991) |
|  |  | *Hyoscyamus niger* L. | *A. rhizogenes* strain 15834 | *p*Ri 15834 | Hot methanol | **—** | Production of hyoscyamine | (Uchida et al., 1993) |
|  |  |  | *A. rhizogenes* strain LBA1334 | **—** | Chloroform:methanol: ammonium hydroxide (15:5:1) | Hairy root culture using bubble-column  bioreactor and a hybrid bubble-column/spray bioreactor | Increased hyoscyamine production | (Jaremicz et al., 2013) |
|  |  |  | *A. rhizogenes* strain C58C1 | *p*VHb-AtHRS1, *p*VHb-RecA, and *p*VHb-CoxIV, | Methanol:ammonia (1:1) | **—** | Enhanced production of hyoscyamine | (Guo et al., 2018) |
|  |  | *Hyoscyamus reticulatus* L. | *A. rhizogenes* strains A7,  15834, A13 and D7 | **—** | Methanol | Elicitation using colchicine and UV-B. | 3.2 fold enhanced production of hyoscyamine | (Zeynali et al., 2016) |
|  |  |  | *A. tumefaciens* strain C58C1, *A. rhizogenes* strains,  LBA9402 and A4 | *p*RiA4 and *p*LAL21 containing *pmt* and *h6h* | 30% Ammonium hydroxide:Ethanol (1:19) | **—** | Enhanced production of hyoscyamine | (Dehghan et al., 2017) |
|  |  |  | *A. rhizogenes* strain A7 | **—** | Chloroform:methanol: ammonium hydroxide (15:5:1) | Elicitation with iron oxide nanoparticles | Five fold increase in hyoscyamine | (Moharrami et al. 2017) |
|  |  |  | *A. rhizogenes* strain A7 | **—** | Chloroform:methanol: ammonium hydroxide (15:5:1) | **—** | Greater productions of hyoscyamine | (Khezerluo et al., 2018) |
|  |  |  | *A. rhizogenes* strain A7 | **—** | Chloroform:methanol: ammonium hydroxide (15:5:1) | Acetylsalicylic acid elicitation | 1.6 fold enhanced production of hyoscyamine | (Norozi et al., 2018) |
|  |  |  | *A. rhizogenes* strain A7 | **—** | Chloroform:methanol: ammonium hydroxide (15:5:1) | **—** | Enhanced production of hyoscyamine | (Asl et al., 2019) |
|  |  |  | *A. rhizogenes* strain A4 and *A. tumefaciens* C58C1 | *p*RiA4-*p*BMI containing *pmt* gene | Chloroform:methanol: ammonium hydroxide (15:5:1) | **—** | Improvement of hyoscyamine | (Shameh et al., 2021) |
|  |  | *Przewalskia tangutica* Maxim. | *A. rhizogenes* strain A4 | **—** | Ethanol:28 % ammonium hydroxide (19:1) | **—** | Enhanced production of hyoscyamine | (Lan and Quan, 2010) |
|  |  | *Scopolia japonica* Maxim. | *A. rhizogenes* strains 15834 | **—** | Ethanol:28 % ammonium hydroxide (19:1) | **—** | Production of hyoscyamine | (Mano et al., 1986) |
|  |  |  | *A. rhizogenes* strains 15834 | **—** | Ethanol:28 % ammonium hydroxide (19:1) | **—** | Production of hyoscyamine | (Nabeshima et al., 1986) |
|  | Isophysalins B | *Physalis angulata* L. | *A. rhizogenes* strain C58C1 | **—** | Methanol | **—** | Increased abundance of isophysalins B | (Zhang et al., 2018) |
|  | Isophysalins G | *Physalis angulata* L. | *A. rhizogenes* strain C58C1 | **—** | Methanol | **—** | Increased abundance of isophysalins G | (Zhang et al., 2018) |
|  | Kukoamine A | *Lycium ruthenicum*  Murr. | *A. rhizogenes* strains ARqua-1,  MSU440 and R1000 | *p*CAMBIA1307-TCP4-OE and *p*CAMBIA1391Z | 0.1% formic acid in 70% methanol | **—** | Higher relative abundances of different secondary metabolites and kukoamine A | (Chahel et al., 2019) |
|  | Lubimin | *Hyoscyamus muticus* L. | *A. rhizogenes* | **—** | C18 sep-pak cartridge, methanol | Elicitation by salicylic acid, ethanol, methyl jasmonate | Increased production of lubimin | (Mehmetoğlu and Curtis, 1996) |
|  |  | *Solanum tuberosum* L. | *A. rhizogenes* | **—** | Methylene chloride/methanol (2:1) | **—** | Enhanced production of lubimin | (Komaraiah et al., 2003) |
|  | Nicotine | *Nicotiana rustica* L. | *A. rhizogenes* strain  LBA9402 | **—** | 2% NH_4_OH, chloroform | **—** | Production of nicotine | (Hamill et al., 1986) |
|  |  |  | *A. rhizogenes* strain  LBA9402 | **—** | 2% NH_4_OH, chloroform | **—** | Improved production of nicotine | (Rhodes et al., 1986) |
|  |  |  | *A. rhizogenes* strain  LBA9402 | **—** | 2% NH_4_OH, chloroform | **—** | Increased production of nicotine | (Robins et al., 1987) |
|  |  |  | *A. rhizogenes* strain  LBA9402 | **—** | 2% NH_4_OH, chloroform | **—** | Production of nicotine | (Furze et al., 1987) |
|  |  |  | *A. rhizogenes* strain  LBA9402 | **—** | 2% NH_4_OH, chloroform | **—** | Production of nicotine | (Benson and Hamill, 1991) |
|  |  | *Nicotiana tabacum* L. | *A. rhizogenes* strain ATCC 15834 | **—** | 5M KOH, CH_2_Cl_2_ | **—** | Production of nicotine | (Zhao et al., 2013) |
|  | Physagulins A | *Physalis angulata* L. | *A. rhizogenes* strain C58C1 | **—** | Methanol | **—** | Increased abundance of physagulins A | (Zhang et al., 2018) |
|  | Physagulins B | *Physalis angulata* L. | *A. rhizogenes* strain C58C1 | **—** | Methanol | **—** | Increased abundance of physagulins B | (Zhang et al., 2018) |
|  | Physagulins C | *Physalis angulata* L. | *A. rhizogenes* strain C58C1 | **—** | Methanol | **—** | Increased abundance of physagulins C | (Zhang et al., 2018) |
|  | Physalin A | *Physalis angulata* L. | *A. rhizogenes* strain C58C1 | **—** | Methanol | **—** | Increased abundance of physalin A | (Zhang et al., 2018) |
|  | Physalin B | *Physalis angulata* L. | *A. rhizogenes* strain C58C1 | **—** | Methanol | **—** | Increased abundance of physalin B | (Zhang et al., 2018) |
|  |  | *Physalis minima* L. | *A. rhizogenes* strain LBA9402 | **—** | Methanol | **—** | Production of physalin B | (Jualang Azlan et al., 2002) |
|  |  | *Physalis minima* L. | *A. rhizogenes* strain LBA9402 | **—** | Methanol | Hairy root culture in shake flask | Increased production of physalin B | (Gansau and Mahmood, 2013) |
|  | Physalin C | *Physalis angulata* L. | *A. rhizogenes* strain C58C1 | **—** | Methanol | **—** | Increased abundance of physalin C | (Zhang et al., 2018) |
|  | Physalin D | *Physalis angulata* L. | *A. rhizogenes* strain C58C1 | **—** | Methanol | **—** | Increased abundance of physalin D | (Zhang et al., 2018) |
|  | Physalin F | *Physalis minima* L. | *A. rhizogenes* strain LBA9402 | **—** | Methanol | **—** | Production of F | (Jualang Azlan et al., 2002) |
|  |  | *Physalis minima* L. | *A. rhizogenes* strain LBA9402 | **—** | Methanol | Hairy root culture in shake flask | Increased production of physalins F | (Gansau and Mahmood, 2013) |
|  |  | *Physalis angulata* L. | *A. rhizogenes* strain C58C1 | **—** | Methanol | **—** | Increased abundance of physalins F | (Zhang et al., 2018) |
|  | Physalin G | *Physalis angulata* L. | *A. rhizogenes* strain C58C1 | **—** | Methanol | **—** | Increased abundance of physalin G | (Zhang et al., 2018) |
|  | Physalin H | *Physalis angulata* L. | *A. rhizogenes* strain C58C1 | **—** | Methanol | **—** | Increased abundance of physalin H | (Zhang et al., 2018) |
|  | Physalin L | *Physalis angulata* L. | *A. rhizogenes* strain C58C1 | **—** | Methanol | **—** | Increased abundance of physalin L | (Zhang et al., 2018) |
|  | Physalin P | *Physalis angulata* L. | *A. rhizogenes* strain C58C1 | **—** | Methanol | **—** | Increased abundance of physalin P | (Zhang et al., 2018) |
|  | Phytuberin | *Solanum tuberosum* L. | *A. rhizogenes* | **—** | Methylene chloride/methanol (2:1) | **—** | Enhanced production of phytuberin | (Komaraiah et al., 2003) |
|  | Phytuberol | *Solanum tuberosum* L. | *A. rhizogenes* | **—** | Methylene chloride/methanol (2:1) | **—** | Enhanced production of phytuberol | (Komaraiah et al., 2003) |
|  | Rishitin | *Solanum tuberosum* L. | *A. rhizogenes* | **—** | Methylene chloride/methanol (2:1) | **—** | Enhanced production of rishitin | (Komaraiah et al., 2003) |
|  | Scopolamine | *Anisodus acutangulus* C.Y.Wu & C.Chen | *A. tumefaciens* strain C58C1 | *p*RiA4 and  *p*CAMBIA1304^+^ carrying *AaTRI* | Chloroform:methanol: ammonium hydroxide (15:5:5) | *AaTRII* gene transformed hairy root culture | 8-fold higher level production of  scopolamine | (Kai et al.. 2009) |
|  |  | *Anisodus luridus* Link | *A. rhizogenes*, strain ATCC 1583 | **—** | **—** | **—** | Production of scopolamine | (Jovanović et al., 1991) |
|  |  |  | *A. tumefaciens* strain C58C1 | *p*RiA4 | Ethanol:28% ammonium hydroxide (19:1) | **—** | Production of scopolamine | (Qin et al., 2014) |
|  |  | *Atropa acuminata* Royle ex Lindl. | *A. rhizogenes* strain A4, LBA9402 | **—** | 5% sulfuric acid (H_2_SO_4_) | Methyl-β-cyclodextrin and coronatine as elicitors | 10 fold enhanced production of scopolamine | (Fattahi et al., 2021) |
|  |  | *Atropa baetica* Willk. | *A. rhizogenes* (strain ATCC 15834) | **—** | Chloroform:methanol: ammonium hydroxide (15:5:1) | **—** | Enhanced production of scopolamine | (Zárate,1999) |
|  |  |  | *A. rhizogenes* strain LBA1334 | *p*BinPlus | Chloroform:methanol: ammonium hydroxide (15:5:1) | Metabolic engineering through overexpression of *h6h* gene | 9 fold increase in scopolamine content | (Zárate et al., 2006) |
|  |  |  | *A. rhizogenes* strain LBA1334 | *p*BinPlus | Chloroform:methanol: ammonium hydroxide (15:5:1) | Elicitation through acetylsalicylic acid, methyl jasmonate | Increased production of scopolamine | (el Jaber-Vazdekis et al., 2008) |
|  |  | *Atropa belladonna* L. | *A. rhizogenes* strain 15834 | *p*Ri 15834 | Chloroform:methanol: ammonium hydroxide (15:5:1) | **—** | Higher productions of scopolamine | (Kamada et al., 1986) |
|  |  |  | *A. tumefaciens* strain  C58C1 | *p*RiA4 containing *pmt* and *h6h* | Ethanol:28 % ammonium hydroxide (19:1) | Overexpression of *pmt* and *h6h* gene | 1.3 - 2.5 fold increase in scopolamine content | (Liu et al., 2010) |
|  |  |  | *A. tumefaciens* strain  C58C1 | *p*RiA4, *p*XI containing *pmt* and *h6h* | Ethanol:28 % ammonium hydroxide (19:1) | Metabolic engineering through overexpression of *pmt* and *h6h* gene | Five fold enhancements in scopolamine production | (Yang et al., 2011) |
|  |  |  | *A. tumefaciens* strain LBA4404 | *p*XI containing *pmt* and *h6h* | Ethanol:28 % ammonium hydroxide (19:1) | Metabolic engineering through overexpression of *pmt* and *h6h* gene | 7.3 fold increase in scopolamine productions | (Wang et al., 2011) |
|  |  | *Brugmansia* × *candida* Pers. | *A. rhizogenes* strain  LBA9402 | **—** | 0.2% H_2_SO_4_, chloroform | Elicitation by hemicellulose, homogenized *Brugmansia candida* roots and CuSO_4_ | Enhanced production of scopolamine | (Pitta-Alvarez and Giulietti, 1998) |
|  |  |  | *A. rhizogenes* strain  LBA9402 | **—** | 0.2% H_2_SO_4_, chloroform | **—** | Elicited productions of scopolamine | (Pitta-Alvarez et al., 2000) |
|  |  |  | *A. rhizogenes* strain  LBA9402 | **—** | 0.2% H_2_SO_4_, chloroform | **—** | Superior productions of scopolamine | (Pitta-Alvarez et al., 2003) |
|  |  | *Datura metel* L. | *Escherichia coli* strain DH5a and *Agrobacterium tumefaciens*  strain C58C1 | *p*MBI and *p*RiA4 | Ethanol: 28% ammonium hydroxide (9:1) | Metabolic engineering through overexpression of *pmt* gene | Enhanced production of scopolamine | (Moyano et al., 2003) |
|  |  |  | *A. rhizogenes* A4 | **—** | Ethanol containing 2% H_2_SO_4_ | Elicitation by *Bacillus cereus* and *Staphylococcus aureus* | Enhanced production of scopolamine | (Shakeran et al., 2017) |
|  |  | *Duboisia leichhardtii* F.Muell. | *A. rhizogenes* strains 15834 and A4 | **—** | Ethanol:28 % ammonium hydroxide (19:1) |  | Increased production of scopolamine | (Mano et al., 1989) |
|  |  |  | *A. rhizogenes* strains 15834 | **—** | Ethanol:28 % ammonium hydroxide (19:1) | Hairy root culture in airlift reactor and column packed with amberlite | Higher production of scopolamine | (Muranaka et al., 1992) |
|  |  |  | *A. rhizogenes* strains 15834 | **—** | Methanol and 28% ammonium hydroxide | **—** | Higher production of scopolamine | (Muranaka et al. 1993) |
|  |  |  | *A. rhizogenes* strains 15834 | **—** | Ethanol:28 % ammonium hydroxide (19:1) | **—** | Production of scopolamine | (Muranaka et al., 1993) |
|  |  |  | *Agrobacterium rhizogenes* strain ATCC 15834 and LBA9402 | *H6H* and *HCHL* | Chloroform:methanol: ammonium hydroxide (15:5:1) | **—** | Production of scopolamine | (ur Rahman et al., 2006) |
|  |  |  | *A. rhizogenes* | *QPT-RNAi* | 0.05 N H_2_SO_4_ | *Quinolinate phosphoribosyl transferase* (*QPT*) gene was silenced using the *QPT-RNAi* construct | Enhanced production of scopolamine | (Singh et al., 2018) |
|  |  | *Duboisia myoporoides* R.Br. | *A. rhizogenes* strain  HRI | **—** | **—** | **—** | Lower production of scopolamine | (Deno et al., 1987) |
|  |  | *Hyoscyamus albus* L. | *A. rhizogenes* strain MAFF 03-01724 | **—** | Chloroform:methanol: ammonium hydroxide (15:5:1) | **—** | Production of scopolamine | (Sauerwein and Shimomura, 1991) |
|  |  | *Hyoscyamus muticus* L. | *A. rhizogenes* strains,  LBA9402 and A4 | **—** | Chloroform:methanol: ammonium hydroxide (15:5:1) | **—** | High level production of scopolamine | (Zolala et al., 2007) |
|  |  |  | *A. rhizogenes* strains,  LBA9402 and ATCC 15834 | **—** | Methanol | Expression of 35S-*h6h* transgene | 100 times more production of scopolamine | (Jouhikainen et al., 1999) |
|  |  | *Hyoscyamus niger* L. | *A. rhizogenes* ATCC 15834 strain | **—** | 0.1 N H_2_SO_4_ | **—** | Production of scopolamine | (Jaziri et al., 1988) |
|  |  |  | *A. tumefaciens* strain  C58C1 | *p*RiA4 containing *pmt* and *h6h* | Ethanol:28 % ammonium hydroxide (19:1) | Overexpression of *pmt* and *h6h* gene | Over nine times higher production of scopolamine | (Zhang et al., 2004) |
|  |  |  | *A. tumefaciens* strain C58C1 | *p*MBI and *p*RiA4 containing *pmt* | Ethanol:28 % ammonium hydroxide (19:1) | **—** | Increased production of scopolamine | (Zhang et al., 2007) |
|  |  |  | *A. rhizogenes* strain LBA1334 | **—** | Chloroform:methanol: ammonium hydroxide (15:5:1) | Hairy root culture using bubble-column  bioreactor and a hybrid bubble-column/spray bioreactor | Increased scopolamine production | (Jaremicz et al., 2013) |
|  |  |  | *A. rhizogenes* strain C58C1 | *p*VHb-AtHRS1, *p*VHb-RecA, and *p*VHb-CoxIV, | Methanol:ammonia (1:1) | **—** | Enhanced production of scopolamine | (Guo et al., 2018) |
|  |  | *Hyoscyamus reticulatus* L. | *A. rhizogenes* strain ATCC 15834 and TR 105 | **—** | 0.2 M H_2_SO_4_ |  | Increased scopolamine production | (Ionkova, 1992) |
|  |  |  | *A. rhizogenes* strains A7,  15834, A13 and D7 | **—** | Methanol | Elicitation using colchicine and UV-B. | 5.1 fold enhanced production of scopolamine | (Zeynali et al., 2016) |
|  |  |  | *A. rhizogenes* strain A7 | **—** | Chloroform:methanol: ammonium hydroxide (15:5:1) | Elicitation with iron oxide nanoparticles | Five fold increase in scopolamine | (Moharrami et al. 2017) |
|  |  |  | *A. rhizogenes* strain A7 | **—** | Chloroform:methanol: ammonium hydroxide (15:5:1) | **—** | Greater productions of scopolamine | (Khezerluo et al., 2018) |
|  |  |  | *A. rhizogenes* strain A7 | **—** | Chloroform:methanol: ammonium hydroxide (15:5:1) | Acetylsalicylic acid elicitation | 3.5 fold enhanced production of scopolamine | (Norozi et al., 2018) |
|  |  |  | *A. rhizogenes* strain A7 | **—** | Chloroform:methanol: ammonium hydroxide (15:5:1) | **—** | Enhanced production of scopolamine | (Asl et al., 2019) |
|  |  |  | *A. rhizogenes* strain A4 and *A. tumefaciens* C58C1 | *p*RiA4-*p*BMI containing *pmt* gene | Chloroform:methanol: ammonium hydroxide (15:5:1) | Overexpression of *pmt* gene | Improvement of scopolamine production | (Shameh et al., 2021) |
|  |  | *Przewalskia tangutica* Maxim. | *A. rhizogenes* strain A4 | **—** | Ethanol:28 % ammonium hydroxide (19:1) | **—** | Enhanced production of scopolamine | (Lan and Quan, 2010) |
|  |  | *Scopolia japonica* Maxim. | *A. rhizogenes* strains 15834 | **—** | Ethanol:28 % ammonium hydroxide (19:1) | **—** | Production of hyoscyamine | (Mano et al., 1986) |
|  |  |  | *A. rhizogenes* strains 15834 | **—** | Ethanol:28 % ammonium hydroxide (19:1) | **—** | Production of scopolamine | (Nabeshima et al., 1986) |
|  | Solasodine | *Physalis minima* L. | *A. rhizogenes* strain ATCC 15834 | **—** | Methanol | **—** | Manifold increased production of solasodine | (Putalun et al., 2004) |
|  |  | *Solanum aviculare* G.Forst. | *A. rhizogenes* strain A4 | **—** | 1N methanolic HCl | **—** | Higher productions of solasodine | (Subroto and Doran, 1994) |
|  |  |  | *A. rhizogenes* strain A4 | **—** | 1N HCl, CHCl_3_ | **—** | Production of solasodine | (Yu et al., 1996) |
|  |  |  | *A. rhizogenes* strain A4 | **—** | 1M methanolic HCl, CHCl_3_ | **—** | Higher productions of solasodine | (Kittipongpatana et al., 1998) |
|  |  |  | *A. rhizogenes* strain LBA9402 | *p*AHM4IS-2 containing HMGR cDNA and one intron sequence | 1M HCl, CHCl_3_ | **—** | Manifold increase in solasodine accumulation | (Argôlo et al., 2000) |
|  |  | *Solanum erianthum* D.Don | *A. rhizogenes* strain A4 | **—** | Methanol | **—** | Increased production of solasodine | (Sarkar et al., 2020) |
|  |  | *Solanum laciniatum* Aiton | *A. rhizogenes* strains 15834 | **—** | 1N HCl, CHCl_3_ | **—** | Production of solasodine | (Okršlar et al., 2002) |
|  |  | *Solanum mammosum*L. | *A. rhizogenes* strains ATCC31798 and A4 | **—** | 96% ethanol | Methyl jasmonate elicitation, cholesterol  and L-arginine precursor feeding | Five times higher production of solasodine | (Ooi et al., 2016) |
|  |  | *Solanum mauritianum* Scop. | *A. rhizogenes* strains LBA 9402, R1600, R1601, TR8/3, A4 and HRI. | **—** | 80% ethanol | **—** | Higher productions of solasodine | (Drewes and Staden, 1995) |
|  |  |  | *A. rhizogenes* strain  LBA 9402 | *p*Ri 1855 and the binary vector pBIN 19 | 1N methanolic HCl | **—** | Increased solasodine production | (Jacob and Malpathak, 2005) |
|  |  | *Solanum myriacanthum* Dunal | *A. rhizogenes* strains A4 and LBA9402 | *p*A4 and *p*1855 | 1N methanolic HCl | **—** | Increased solasodine production | (Jacob and Malpathak, 2005) |
|  |  |  | *A. rhizogenes* strain A4 | **—** | Ethanol | Elicitation through NaCl | Manifold increase in solasodine production | (Srivastava et al., 2016) |
|  |  | *Solanum trilobatum* L. | *A. rhizogenes* strain 532, 2364, A4 and R1000 | **—** | 1N HCl, CHCl_3_ | Methyl jasmonate elicitation | 1.9 fold elevated production of solasodine | (Shilpha et al., 2015) |
|  |  | *Solanum virginianum* L. | *A. rhizogenes* strain MTCC 2364 and 532 | **—** | 25 % ammonia and ethanol (1:1), Methanol | **—** | Production of solasodine | (Pawar et al., 2008) |
|  | Solavetivone | *Hyoscyamus albus* L. | *A. rhizogenes* strain MAFF 03-01724 | **—** | Ethyl acetate | Methyl jasmonate and CuSO_4_ elicitation | Production of solavetivone | (Kuronayagi et al., 1998) |
|  |  | *Hyoscyamus muticus* L. | *A. rhizogenes* | **—** | C18 sep-pak cartridge, methanol | Elicitation by salicylic acid, ethanol, methyl jasmonate | Increased production of solavetivone | (Mehmetoğlu and Curtis, 1996) |
|  | Tropane alkaloids | *Anisodus acutangulus* C.Y.Wu & C.Chen | *A. tumefaciens* C58C1 strain | *p*CAMBIA1304^+^ containing *AaPMT* and *AaTRI* | 95% ethanol and water | Coexpression of *AaPMT* and *AaTRI* genes | 8.66 fold increased production of tropane alkaloids | (Kai et al., 2011) |
|  |  |  | *A. tumefaciens* strain C58C1  containing *A. rhizogenes* Ri plasmid | *p*CAMBIA1304^+^ containing *AaTRI* and *AaH6H* | Chloroform:methanol: ammonium hydroxide (15:5:5) | Metabolic engineering through overexpression of *TRI* and *H6H* gene | 4.49 fold increased production of tropane alkaloids | (Kai et al., 2012) |
|  |  |  | *A. tumefaciens*  strain C58C1 | *p*RiA4 | Chloroform:methanol: ammonium hydroxide (15:5:5) | Elicitation using ethanol, methyl jasmonate and Ag^+^ | Improvements of tropane alkaloids production | (Kai et al., 2012) |
|  |  |  | *A. rhizogenes* strains 15834 | **—** | Methanol, 2% H_2_SO_4_ | Hairy root culture in stirred-tank reactor | Production of tropane alkaloids | (Marchev et al., 2012) |
|  |  |  |  | **—** | **—** | **—** | Enhanced production of tropane alkaloids | (Liu et al., 2013) |
|  |  | *Anisodus tanguticus* (Maxim.) Pascher | *A. rhizogenes* strain ATCC10060 (A4), MSU440, ATCC11325 (LBA9402) | *p*RiA4 and *p*Ri1855 | Chloroform:methanol: ammonium hydroxide (15:5:1) | Hairy root culture in flask reactor | Enhanced production of tropane alkaloids | (Lei et al., 2020) |
|  | Withaferin A | *Physalis minima* L. | *A. rhizogenes* strain A4 | **—** | Methanol | **—** | Enhanced production of withaferin A | (Halder and Ghosh, 2022) |
|  |  | *Withania coagulans* (Stocks) Dunal | *A. tumefaciens* C58C1 | *p*RiA4 | Methanol:water (25:75) | **—** | Production of withaferin A | (Mirjalili et al., 2009) |
|  |  | *Withania somnifera* (L.) Dunal | *A. rhizogenes* strains 15834, R1000, K599 | **—** | Methanol | **—** | Accumulation of withaferin A | (Saravanakumar et al., 2012) |
|  |  |  | *A. rhizogenes* strains 15834, A4, LBA9402 and *A. tumefaciens* C58C1 | **—** | Methanol | Elicitation by Methyl jasmonate and β-cyclodextrin | Up to 12.46 fold increase in withaferin A content | (Karami et al., 2023) |
|  |  |  | *Agrobacterium rhizogenes* strain R1000 | **—** | Methanol | Salicylic acid elicitation | 42 fold higher production of withaferin A | (Sivanandhan et al., 2013) |
|  | Withangulatin A | *Physalis angulata* L. | *A. rhizogenes* strain C58C1 | **—** | Methanol | **—** | Increased abundance of withangulatin A | (Zhang et al., 2018) |
|  | Withanolide A | *Withania coagulans* (Stocks) Dunal | *A. tumefaciens* C58C1 | *p*RiA4 | Methanol:water (25:75) | **—** | Production of withanolide A | (Mirjalili et al., 2009) |
|  |  |  | *A. tumefaciens* C58C1 | *p*RiA4 and/or *p*BIs *SS1* | Methanol:water (25:75) | **—** | Enhanced production of withanolide A | (Mirjalili et al., 2011) |
|  |  | *Withania somnifera* (L.) Dunal | *A. rhizogenes* strain R1601 | **—** | Methanol | **—** | Enhanced production of withanolide A | (Praveen and Murthy, 2003) |
|  |  |  | *Agrobacterium rhizogenes* strain R1000 | **—** | Methanol | Salicylic acid elicitation | 58 fold higher production of withanolide A | (Sivanandhan et al., 2013) |
|  | Withanolides | *Withania coagulans* (Stocks) Dunal | *A. rhizogenes* strain A4 | **—** | **—** | **—** | Enhanced production of withanolides | (Mirjalili et al., 2009) |
|  |  |  | *Agrobacterium rhizogenes* strain R1000 | **—** | Methanol | Higher expression of squalene synthase gene | 1.08 to 1.25-times higher production of withanolides | (Sivanandhan et al., 2020) |
|  | Withanone | *Withania somnifera* (L.) | *Agrobacterium rhizogenes* strain R1000 | **—** | Methanol | Salicylic acid elicitation | 46 fold higher production of withanone | (Sivanandhan et al., 2013) |
| **^⁕^** “—“ Not mentioned in the respective article | | | | | | | | |
